# Social determinants of adult mortality from non-communicable diseases in northern Ethiopia, 2009-2015: Evidence from health and demographic surveillance site

**DOI:** 10.1371/journal.pone.0188968

**Published:** 2017-12-13

**Authors:** Semaw Ferede Abera, Alemseged Aregay Gebru, Hans Konrad Biesalski, Gebisa Ejeta, Andreas Wienke, Veronika Scherbaum, Eva Johanna Kantelhardt

**Affiliations:** 1 Institute of Biological Chemistry and Nutrition, University of Hohenheim, Stuttgart, Germany; 2 Food Security Center, University of Hohenheim, Stuttgart, Germany; 3 School of Public Health, College of Health Sciences, Mekelle University, Mekelle, Ethiopia; 4 Kilte Awlaelo- Health and Demographic Surveillance Site, Mekelle, Ethiopia; 5 Department of Agronomy, Purdue University, West Lafayette, Indiana, United States of America; 6 Institute of Medical Epidemiology, Biostatistics, and Informatics, Faculty of Medicine, Martin-Luther University, Halle, Germany; 7 Department of Gynaecology, Faculty of Medicine, Martin-Luther University, Halle, Germany; Leibniz Institute for Prvention Research and Epidemiology BIPS, GERMANY

## Abstract

**Introduction:**

In developing countries, mortality and disability from non-communicable diseases (NCDs) is rising considerably. The effect of social determinants of NCDs-attributed mortality, from the context of developing countries, is poorly understood. This study examines the burden and socio-economic determinants of adult mortality attributed to NCDs in eastern Tigray, Ethiopia.

**Methods:**

We followed 45,982 adults implementing a community based dynamic cohort design recording mortality events from September 2009 to April 2015. A physician review based Verbal autopsy was used to identify the most probable causes of death. Multivariable Cox proportional hazards regression was performed to identify social determinants of NCD mortality.

**Results:**

Across the 193,758.7 person-years, we recorded 1,091 adult deaths. Compared to communicable diseases, NCDs accounted for a slightly higher proportion of adult deaths; 33% vs 34.5% respectively. The incidence density rate (IDR) of NCD attributed mortality was 194.1 deaths (IDR = 194.1; 95% CI = 175.4, 214.7) per 100,000 person-years. One hundred fifty-seven (41.8%), 68 (18.1%) and 34 (9%) of the 376 NCD deaths were due to cardiovascular disease, cancer and renal failure, respectively. In the multivariable analysis, age per 5-year increase (HR = 1.35; 95% CI: 1.30, 1.41), and extended family and non-family household members (HR = 2.86; 95% CI: 2.05, 3.98) compared to household heads were associated with a significantly increased hazard of NCD mortality. Although the difference was not statistically significant, compared to poor adults, those who were wealthy had a 15% (HR = 0.85; 95% CI: 0.65, 1.11) lower hazard of mortality from NCDs. On the other hand, literate adults (HR = 0.35; 95% CI: 0.13, 0.9) had a significantly decreased hazard of NCD attributed mortality compared to those adults who were unable to read and write. The effect of literacy was modified by age and its effect reduced by 18% for every 5-year increase of age among literate adults.

**Conclusion:**

In summary, the study indicates that double mortality burden from both NCDs and communicable diseases was evident in northern rural Ethiopia. Public health intervention measures that prioritise disadvantaged NCD patients such as those who are unable to read and write, the elders, the extended family and non-family household co-residents could significantly reduce NCD mortality among the adult population.

## Introduction

Non-communicable diseases (NCDs) are medical conditions or diseases that are, by definition, non-infectious and non-transmissible between people [1]. Major NCDs include cardiovascular diseases, cancer, chronic respiratory diseases, diabetes, rheumatologic diseases and mental disorders, which contribute to about 80% of all NCD-attributed deaths [1–4].

Studies have demonstrated that the burden and sequelae of NCDs are increasing considerably worldwide [5–10], and that NCDs constitute a quadruple burden in developing countries in addition to communicable, neonato-maternal and nutritional diseases, injuries and road traffic accidents [11–13]. NCDs account for the largest share of mortality globally [14, 15], and 82.4% of the total deaths occur in low and middle income countries [15].

In recent years, global attention and the commitment to address NCDs has grown rapidly. Sustainable Development Goal 3 (SDG 3) target 3.4 [16] of the WHO states “By 2030, reduce by one third premature mortality from NCDs through prevention and treatment and promote mental health and well-being” to ensure that global development is sustanable [17–19]. If health policies are not shaped by reliable evidence for the purpose of optimizing disease interventions, the achievement of this target will likely be an ambition particualrly in low and middle income countries (LMICs) due to the fact that health sectors in LMICs are also strained by the existence of other resource-intensive competing priorities [13]. In 2012, a study conducted by Weldearegawi et al. in northern Ethiopia, from the same study area, indicated that NCDs accounted for 28.6% of the total deaths [20]. However, the aim of the study was merely descriptive and did not attempt to inferentially identify the factors associated with NCD-attributed mortality.

Health or illness status of individuals are determined by social structures through micro level processes, and the quality of these social structures are in turn shaped by macro level processes like the political ideology of governments [21]. Nowadays, effective reduction in population and individual level health inequality should be viewed from the philosophical and holistic perspective of integrating interventions that could address social and economic inequalities between and within population [21–25].

According to the World Health Organization (WHO)’s Commission on Social Determinants of Health analytical framework for priority public health conditions, social and economic factors have a vital public health importance. These factors influence exposure to several downstream factors, vulnerability to disease development, utilization of health care services, health outcomes and consequences [26].

Several studies have consistently showed that attaining higher education is observed as the strongest indicator of social determinant of good health [27–30]. Low educational attainment was reported to predict increased risk for and mortality from NCDs [31–38], low longevity [39, 40], and poor health service utilization [40, 41]. The consistently strong association of education with the risk of developing NCDs and dying from NCDs, even after controlling the effect of lifestyle factors, may indicate its causal relationship [28, 31, 33]. In one large cohort study, having a less educated partner was associated with higher risk of mortality [42]. On the other hand, studies have shown that unemployed individuals had higher NCD risk factor profile [43], differential odds of developing and dying from some NCDs and all-cause mortality [44–46], and these effects remained persistent after adjusting for the use of preventive medications, clinical and lifestyle factors. In addition, employment insecurity was also associated with a decrease in preventive health service utilization [47].

The relationship of income inequality and poor health was extensively studied [48, 49]. Higher risks of morbidity and mortality from NCDs were also associated with income status [50–53]. However, the adverse effect of income inequality on health becomes more pronounced if the inequality exists at national level than in small areas [54]. In the literature, three main hypotheses (social capital, status anxiety and neo-materialist hypotheses) are widely mentioned to explain the mechanisms income inequality follows to result in poor health [21, 49]. Some other studies, however, showed that wealth status may not be an important factor in relation to mortality for some NCDs like acute myocardial infarction [55]. Several research works have also established that household composition and structure, marital status, age and ethnicity could be germane factors in epidemiological investigations focusing on mortality [7, 32, 40, 56–60]. In general, the mechanisms of the socio-economic and demographic determinants of NCD attributed mortality are shown in the conceptual framework customized from the WHO’s Commission on Social Determinants of Health analytical framework for priority public health conditions [26], as described in the supplementary document (S1 Fig).

Evidence on social determinants of NCD attributed mortality is essential for evidence based public health actions against NCDs. Despite such importance, to the best of our knowledge, there has been no detailed systematic quantification of the effect of such factors on the risk of mortality from NCDs using population-based prospective data in Ethiopia. Consequently, our understanding of how socio-demographic and economic determinants drive NCD-attributed mortality is very limited.

Bearing these factors in mind, the present paper presents the adult mortality burden due to NCDs and its socio-demographic and economic determinants in northern Ethiopia using population-based data from Kilte Awlaelo-Health and Demographic Surveillance Site (KA-HDSS). A description of the broad and specific causes of death by the study population’s characteristics is also provided.

## Methods

### Study setting

Complete civil registration and vital statistics (CRVS) systems provide essential information for public health policy and prevention [61, 62]. Nevertheless, complete CVRS is absent in sub-Saharan Africa including Ethiopia. In countries where CVRS is absent, an alternative effective way of generating reliable and accurate vital events data, although not fully representative, could be made through health and demographic surveillance systems (HDSS)[62]. HDSS collect data on vital events, causes of death, and socio-economic data based on a geographically defined population ranging from tens of thousands to around a quarter of million [63]. Such longitudinal research systems have been implemented in Africa, Asia and Oceania and have been jointly organized by a cross-continental organization called The International Network of field sites with continuous Demographic Evaluation of Populations and Their Health in developing countries (INDEPTH Network) [63]. Under this research network, there are over 3.8 million surveillance population, and KA-HDSS is one of the contributing member sites of this organization [64].

Our current study used data from KA-HDSS, which is located 835 kilometres (KMs) north of Addis Ababa, the capital city of Ethiopia. KA-HDSS was established in 2009 home-based under the School of Public Health in Mekelle University. It is led by the university’s Research and Community Services Directorate and College of Health Sciences with the central research motivation of generating population-based health and demographic-related longitudinal information to support evidence-based decision making. It started its surveillance by enumerating and recording data from 66,438 individuals who were living in 14,453 households. These households were located in 10 kebelles, and only one of the 10 kebelles is urban. According to the Ethiopian government administrative structure, kebelles are the smallest administrative units with an average population of 5,000–6,000.

KA-HDSS collects data on vital events (birth, death, and migration in and out), pregnancy outcome, causes of death and marital status, which change on a continuous basis. Moreover, update data are collected biannually from the entire population and accordingly the surveillance database is updated twice a year. The data collectors are fulltime workers, from among the community, who are intensively trained on the different data collection tools, skills and preparations for interviewing by the research team academics. In addition, refresher training of a similar content is delivered to the data collectors twice a year before the start of each update. Generally, the study area is characterised by low fertility and mortality [20, 60] and high external outmigration of adults to the Arab world.

### Verbal autopsy data

Information on mortality and its causes is critical to public health planning and action. Such public health information is non-existent in developing countries where complete vital registration is not available and most deaths occur at home [65]. Collecting cause-specific mortality information using the verbal autopsy (VA) method is the most feasible, comprehensive and appropriate approach to narrow mortality information gaps in resource-limited settings [66–68]. VA is a research tool used to assign probable causes of death by interviewing a close caregiver about the signs and symptoms of the deceased’s terminal illness [68].

In KA-HDSS, mortality data are collected from the closest adult care givers who took care of the deceased individuals, within 45–55 days after each death event, considering the mourning period of the community, by trained high school completed residents. The surveillance research project collects VA data using standard VA questionnaires for neonates, post-neonates and children and adults [69]. The current study used the VA data set for adults, whose age was greater than 15 years at the time of death, collected from September 2009 to April 2015.

We applied the physician review method to identify the most probable cause of death. The reviewing physicians are trained for the use of International Classification of Diseases (ICD-10) [69]. The collected questionnaire-based VA data were submitted to two blinded physicians for their independent review to assign the probable cause of death. If the two physicians concluded a specific cause of death, say tuberculosis (TB), then the cause of death was considered to be TB. If the two blinded physicians assigned two different causes of death, then a third blinded physician was invited to review and assign a cause of death which served as a tie breaker. If the third physician assigned a cause of death different from assigned causes by either of the first two physicians, the cause of death was classified as “indeterminate”.

In this study, premature mortality from NCDs was defined as the relative proportion of NCD death in the 15–64 age group. The cut-off of age 65 years was used in accordance with the estimated life expectancy for Ethiopia [15]. A similar definition was also used by Streatfield et al. [58]. Moreover, in each household, one person was defined as the head of a given household. In the context of the study area, the husband is usually the head of the household. If the husband is dead, then the husband’s wife, the mother, is usually the head of the household. The relationship of household members to the household head can be either close or extended family members or non-family member. Geographic locations of households, derived from the altitudinal measurements obtained using GPS, were classified as located in midland or highland.

The WHO defines social determinants of health as “The conditions in which people are born, grow, work, live, and age, and the wider set of forces and systems shaping the conditions of daily life. These forces and systems include economic policies and systems, development agendas, social norms, social policies and political systems” [70]. In this study, social determinants such as education, occupation, marital status, residence, geographic location, wealth index, intra-household relationships, household headship, sex of household head, health seeking practice during terminal illness were considered. The effects of demographic factors (age and sex) were also examined in the analysis. Data on these variables were collected using KA-HDSS data collection tools.

### Data management and statistical analysis

The dataset was cleaned and checked for the plausibility of the data values. Principal component analysis (PCA) was used to generate wealth index variable reproducing the principles and methods of Demographic and Health Survey (DHS) [71]. The wealth index variable was constructed from the attributes of goods and resources owned by the households (for instance, ownership of radio/tape), construction material and characteristics of the houses (types of floor, roof and wall), and access to resources and services (like access to electricity, land ownership, size of land owned as measured in hectare, drinking water sources, toilet availability, functionality and type). Indicator variables, coded as 0 for “No” or 1 for “Yes”, were computed from each category of the included categorical variables before PCA was applied. However, the dummied variables that showed no variation were excluded from the analysis. Socio-economic data were not collected for the households which were established after the start of the surveillance because such data should be collected at baseline and every 5 year from the baseline. Consequently, the wealth status of the adult cohort members (18.9%) who lived in those households was unknown. The main outcome was time to death from NCDs and the incidence density was calculated by dividing the number of new NCD attributed mortality events by the person-years of all observations that were at risk of dying from NCDs. Causes of death were classified using the international disease classification system [69]. Accordingly, NCD related mortality includes all adult deaths caused by neoplasms, nutritional and endocrine disorders, diseases of the circulatory system, respiratory disorders, gastrointestinal disorders, renal disorders, mental and nervous system disorders which have VA codes of VA-02, VA-03, VA-04, VA-05, VA-06, VA-07, and VA-08, respectively. Additionally, VA-01 and VA-11 were classified as communicable diseases and external causes of death, respectively. All deaths which had the code VA-09 were classified as pregnancy-, childbirth- and puerperium-related deaths.

The starting point of the surveillance project was 11 September 2009. Entry of new members into the surveillance project was possible by enumeration, external immigration or birth. Corresponding dates for these initiating events were accurately recorded. Moreover, the end point of this study was 26 April 2015. Exit of residents from the surveillance project was possible by outmigration and death. Entries of new members into the cohort population or exits of existing members could happen at any time. So, epidemiologically, this study followed a community based dynamic cohort study design. In such design contexts, the length of the follow-up time contributed by each adult could not be the same due to different enrolment time, censoring or competing interests [72], but the time contribution by each cohort member, measured per person-unit time, should be taken into consideration and must be accurately quantified. The 376 NCD-attributed deaths, of the total 45, 982 cohort members, were events of our interest whereas outmigration and mortality from non-NCD causes were treated as censored cases. The exact dates for these censored events were also recorded. Moreover, all of the observed cases that were alive by 26 April 2015 were also considered as administratively censored cases. More than a quarter, 28.7%, of the adults were externally out-migrated, and these adult residents were loss to follow-up cases.

Cox’s proportional hazards regression model was used to analyse the survival-type data thatinclude censored observations [72, 73]. A Log rank test was used to assess the equality of the survival patterns. The incidence density rates of NCD-attributed mortality and 95% confidence intervals were calculated. A key assumption of the Cox proportional hazards regression model is the proportional hazards assumption which claims that the effect of a given a covariate, quantified in hazard ratio, does not change over time [73–75]. Adherence to this assumption was checked using the plots of scaled and smoothed Schoenfeld residuals with the reference value of zero slope along the survival time [74,75]. Hence, each fitted covariate had a plot approximately parallel to the reference line, a line that depicted a zero slope with no significant departure, proving that the proportional hazards assumption of the model is fulfilled. In order to build a more pragmatic model with improved inference, interaction effects were also checked by forming a set of biologically plausible interaction terms from the main effects in the model. The significance of each separate interaction was assessed by adding each interaction term to the main effects model and using the partial likelihood ratio test [73]. Assessment of the problem of multicollinearity was checked at variance inflation factor (VIF) of greater than 10 and multicollinear variables were dropped from fitting into the model [76].

All of the variables with a p-value less than 0.25 in the univariate analysis were eligible for inclusion into the multivariable Cox proportional hazards regression [73]. Adjusted hazards ratio (HR) was used to estimate the degree of independent effect of each fitted predictor on the time to NCD attributed death. We also performed a sensitivity analysis to assess the impact of lost to follow-up on our estimates and generally, we found no difference in the results (S1 Table). All the analyses were performed in stata 13.0.

### Ethical statement

The Ethiopian Science and Technology Agency has ethically approved KA-HDSS with the identification number IERC-0030. Moreover, an ethical approval of the consent procedure was also obtained from the Health Research Ethics Review Committee (HRERC) in Mekelle University. Since a large proportion of the study population had no formal education, verbal consent was preferred to appropriately inform the interviewee. In addition, the VA data collectors were trained on how to implement the verbal consent and they collected the data from the primary caregivers of the deceased individuals only if they consented for the interview. KA-HDSS data is also highly protected to avoid potential access by third parties. For this purpose, the target dataset was extracted de-identifying personal identifiers to make that the dataset anonymous and maintain the confidentiality of the study participants.

## Results

### Characteristics of the study population and incidence of NCD mortality

This study was based on 45,982 adult KA-HDSS cohort members who contributed 193,758.5 person-years. The population resided in 16,247 households and the mean number of persons per household, including children, was 4. More than four-fifths (n = 37,549; 81.7%), and about three quarters, (n = 32,962; 74.5%), of the population were living in rural residences and midland geographic locations, respectively.

The median age was 32 years, with an interquartile range of 24 years. The age-dependency ratio was 14 per 100 working-age population. Although 53% of the external out-migrants were female, the female population was 3.6% higher than the male population.

As shown in Table 1, about half of the population (n = 22,928; 49.9%) were unable to read and write and more than one third (n = 17,701; 38.5%) were unemployed. More than a third (n = 16,247; 35.4%) and a quarter (n = 13, 350; 29%) of the participants were heads of households and economically poor, respectively (Table 1).

**Table 1:** Socio-demographic, economic and geographic characteristics and NCD deaths per 100,000 person-years of adult KA-HDSS cohort members from September 2009 to April 2015, northern Ethiopia (n = 45,982; 376 NCD deaths).

Characteristics	Frequency by sex		Total, n (%)	person-years	NCDs attributed deaths	IDR (95% CI)
	Female	Male				
**Age**						
15–49 years	18,747	15,944	34,691 (75.4)	138,249.8	63	45.6 (35.6, 58.3)
50–64 years	3,163	2,604	5,767 (12.5)	29,251.6	67	229.1 (180.3, 291)
≥65 years	2,715	2,809	5,524 (12.0)	26,257.3	246	936.9 (826.8, 1061.6)
**Marital status**						
Married	9,728	11,054	20,782 (45.2)	81,081.8	53	65. 4 (49.9, 85.6)
Single	10,354	9,552	19,906 (43.3)	91,207.8	187	205 (177.7, 236.6)
Widowed	2,214	409	2,623 (5.7)	9,732.2	28	287.7 (198.7, 416.7)
Divorced	2,320	334	2,654 (5.8)	11,694.0	104	889.3 (733.8, 1077.8)
**Education**						
Unable to read and write	13,566	9,362	22,928 (49.9)	107,829.8	312	289.4 (259, 323.3)
Primary	6,773	7,298	14,071 (30.6)	57,578.7	44	76.4 (56.9, 102.7)
Secondary and above	4,286	4,697	8,983 (19.5)	28,350.1	20	70. 6 (45.5, 109.4)
**Occupation**						
Unemployed	8,110	9,591	17,701 (38.5)	57,258.9	19	33.2 (21.2, 52)
Farmer	3,515	7,880	11,395 (24.8)	55,555.0	247	444.6 (392.5, 503.7)
Government employee and Others	13,000	3,886	16,886 (36.7)	80,944.9	110	135.9 (112.7, 163.8)
**Residence**						
Rural	19,509	18,040	37,549 (81.7)	167,039.8	331	198.2 (177.9, 220.7)
Urban	5,116	3,317	8,433 (18.3)	26,718.9	45	168.4 (125.8, 225.6)
**Wealth index**						
Poor	7,494	5,856	13,350 (29.0)	57,273.7	109	190.3 (157.7, 229.6)
Medium	4,152	3,759	7,911 (17.2)	37,969.6	90	237 (192.8, 291.4)
Wealthy	7,855	8,166	16,021 (34.8)	78,188.2	146	186.7 (158.7, 219.6)
Unknown	5,124	3,576	8,700 (18.9)	20,327.2	31	152.5 (107.3, 216.9)
**Relation to household head**						
Head of household	5,972	10,275	16,247 (35.4)	69,699.5	237	340 (299.4, 386.2)
Wife/children	17,079	9,951	27, 030 (58.8)	115,365.6	66	57.2 (45, 72.8)
Extended and non- family members	1,565	1,115	2,680 (5.8)	8,622.5	68	788.6 (621.8, 1000.2)
**Household headship**						
No	18,653	11,082	29,735 (64.7)	124,059.2	139	112 (94.9, 132.3)
Yes	5,972	10,275	16,247 (35.3)	69,699.5	237	340 (299.4, 386.2)
**Sex of household head**						
Female	5,972	0	5,972 (36.8)	23,420.7	84	358.7 (289.6, 444.2)
Male	0	10,275	10,275 (63.2)	46,278.8	153	330.6 (282.2, 387.4)
**Geographic location**						
Midland	17,599	15,363	32,962 (74.5)	140,823.7	267	189.6 (168.2, 213.8)
Highland	6,016	5,252	11,268 (25.5)	50,330.5	107	212.6 (175.9, 257)
**Total**	24,625	21,357	45,982 (100)	193,758.7	376	194.1 (175.4, 214.7)

CI: Confidence interval, IDR: Incidence density rate, NCDs: Non-communicable diseases.

Across 193,758.7 person-years, the incidence density rate (IDR) of NCD mortality was 194 deaths (IDR = 194.1; 95% CI = 175.4, 214.7) per 100,000 person-years. The IDR of NCD mortality increased as age increased. The rate was 937 NCD deaths per 100,000 person-years for those who were 65 years and above. The IDR ratio of NCD mortality between 15–64 years to over 64 years was 1:12. On the other hand, the proportion of NCD deaths in the 15–64 years age group, which is interpreted as premature mortality from NCD, was 32.3%. There was a differential occurrence of NCD attributed mortality IDR with most of the other socio-demographic characteristics (Table 1).

Mortality rate of cancer, 37.4 vs. 32.1 deaths per 100,000 person-years, and cardiovascular diseases (CVDs), 83.6 vs. 78.7 deaths per 100,000 person-years, was slightly higher for males compared to females. Contrary to this, the mortality rate of renal failure was higher for females compared to males, 19 vs. 14 deaths per 100,000 person-years, respectively.

### Broad causes of death

During the surveillance period, a total of 1,091 adult deaths were observed. About four-fifths (n = 872; 79.9%) of those died at home. Moreover, causes of death were assigned for 908 (83.2%) deaths. Of the total deceased cases, NCDs accounted for more than one-third (n = 376; 34.5%) of all deaths. Comparable to NCDs, communicable diseases caused 360 (33%) deaths. The third leading causes of death were deaths from external causes (n = 126; 11.5%) (Table 2).

**Table 2:** Broad causes of death, stratified by age and sex, among adult KA-HDSS cohort members from September 2009 to April 2015, northern Ethiopia.

Broad category of cause of deaths	Frequency by sex, n (%)		Frequency by age group, n (%)			Total, n (proportion; 95% CI)
	Female	Male	15–49 years	50–64 years	≥65 years	
Non-communicable diseases	182 (48.4)	194 (51.6)	63 (16.8)	67 (17.8)	246 (65.4)	376 (34.5; 31.7–37.2)
Communicable diseases	194 (53.9)	166 (46.1)	75 (20.8)	59 (16.4)	226 (62.8)	360 (33.0; 30.3–35.8)
External causes of death	34 (27.0)	92 (73.0)	67 (53.2)	15 (11.9)	44 (34.9)	126 (11.5; 9.8–13.6)
Unspecified causes of death	24 (61.5)	15 (38.5)	2 (5.1)	3 (7.7)	34 (87.2)	39 (3.6; 2.6–4.9)
Other causes (pregnancy, child birth and puerperium related causes)	7 (100)	0 (0.0)	7 (100.0)	0 (0.0)	0 (0.0)	7 (0.6; 0.3–1.3)
Undetermined causes	104 (56.8)	79 (43.2)	36 (19.7)	11 (6.0)	136 (74.3)	183 (16.8; 14.7–19.1)
Total deaths	544 (49.9)	547 (50.1)	248 (22.7)	155 (14.2)	688 (63.1)	1,091

CI: Confidence interval.

Nearly two-thirds of the NCD attributed deaths were among those who were 65 years and above. Observably, deaths from communicable diseases were higher by about 4% for females, but about three-fourths of the deaths from external causes occurred among males. In addition, nearly three-fourths of the undetermined causes and 87.2% of the unspecified causes were among those age 65 years and above (Table 2).

### Specific NCD types as causes of death

**Fig 1:**
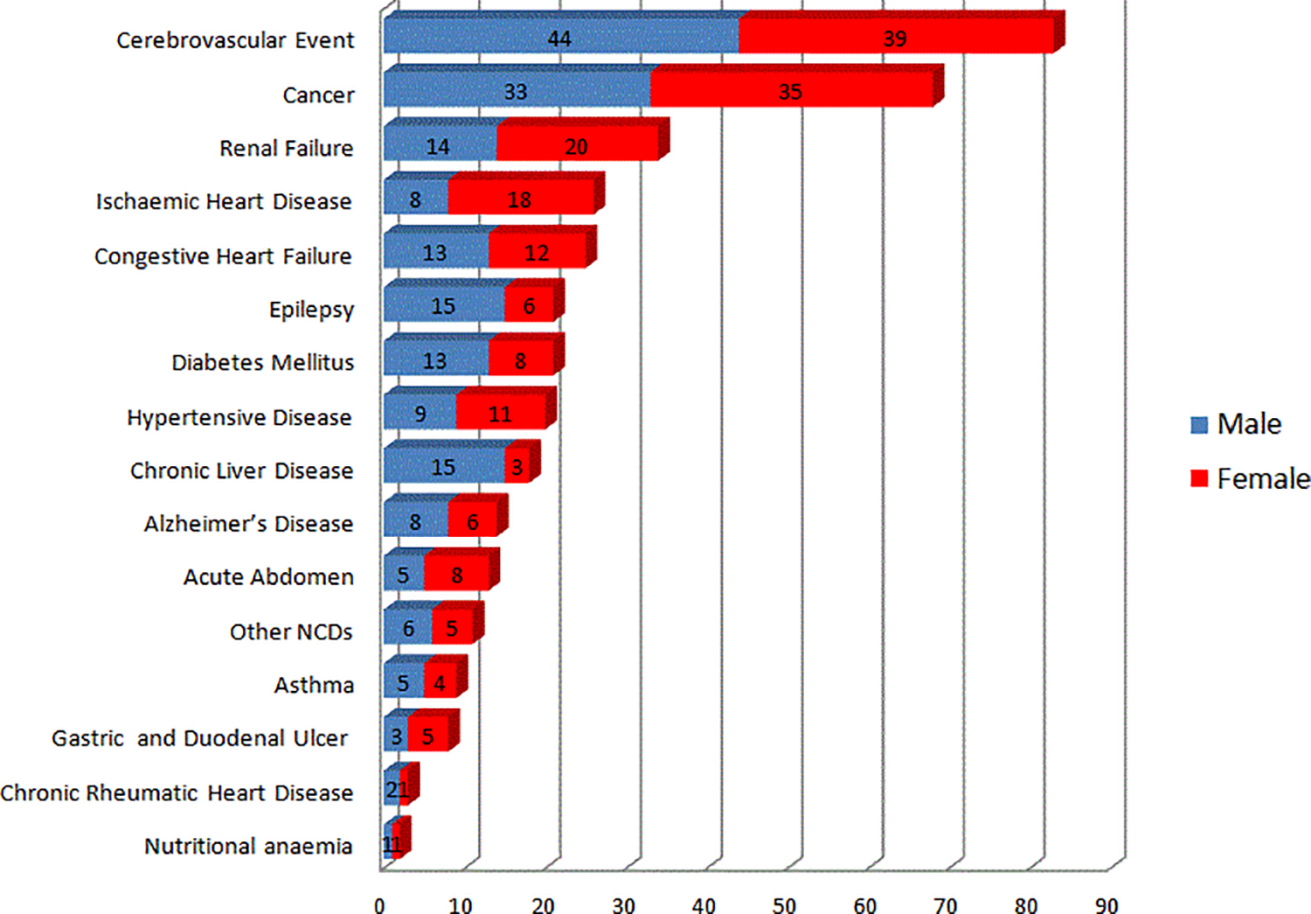
The absolute number of deaths by specific NCD types and sex among the adult KA-HDSS cohort members from September 2009 to April 2015, Tigray region, northern Ethiopia (n = 376).

Of the 376 NCD deaths, cerebrovascular event, cancer and renal failure were the leading causes of adult death, accounting for 83 (22.1%), 68 (18.1%) and 34 (9%) deaths, respectively. Moreover, 6.9% and 6.6% of the total NCD deaths were shared by ischaemic heart disease and congestive heart failure, respectively. We also found that diabetes mellitus, epilepsy, hypertensive disease, chronic liver disease and Alzheimer’s disease were the next top NCDs causing high numbers of death in the KA-HDSS adult population (Fig 1). It is particularly important to mention that cardiovascular diseases (cerebrovascular event, ischaemic heart disease, congestive heart failure, hypertension and chronic rheumatic heart disease) accounted for 41.8% of all NCD-attributed mortality. Furthermore, gastrointestinal cancers contributed more than half (n = 37; 54.4%) of the deaths caused by all types of cancer. Of the total deaths due to all cancer types, for both men and women, nearly half of the deaths (n = 33; 48.5%) were caused by oesophageal cancer (22.1%), stomach cancer (13.2%), and malignant neoplasm of the large and small intestines (13.2%). Moreover, differential mortality of specific NCDs was observed by sex. Generally, listed according to their rank, chronic liver disease (83.3%), epilepsy (71.4%), diabetes mellitus (61.9%), Alzheimer’s disease (57.1%), cerebrovascular events (53%) and congestive heart failure (52%) were more common causes of death among male adults, whereas renal failure, ischaemic heart disease (69.2%), gastric and duodenal ulcer (62.5%), renal failure (58%), hypertensive disease (55%) and cancer (51.5%) were more frequent causes of death among female adults (Fig 1).

### Predictors of NCD attributed mortality

Age, sex, marital status, education, occupation, wealth index, relation to household head and the interaction of education with age were included in the multivariable Cox proportional hazards regression model. The variables residence and geographic location were not accounted for in the multivariable model because these two variables were not significant in the bivariate analysis. Sex of head of the household was also not fitted in to the model because it created a severe multicollinearity problem with the relation to household variable. Therefore, interpretation of the model was done considering the controlled effect of the remaining seven variables in addition to the interaction of age and education.

Age, education, relation to head of household and the interaction of education with age were significantly associated with NCD attributed mortality.

There was a 35% excess hazard of mortality from NCDs for every 5-year increase of age. The mortality hazard from NCDs was also significantly different by educational status. Compared to those who were unable to read and write, the hazard of NCD attributed mortality was 65% lower for those who were literate. However, the reduced NCD mortality for literate individuals was significantly modified by age in that the observed positive effect was reduced by 18% for every 5-year increase in the age of the cohort.

Another striking finding was that extended family and other non-family members, such as servants, had a 2.9 times higher hazard of NCD attributed mortality compared to their respective head of household (Table 3).

**Table 3:** Predictors of NCD mortality among adult KA-HDSS cohort members from 11 September 2009 to 26 April 2015, northern Ethiopia (n = 45,982).

Variables	Crude HR (95% CI)	Adjusted HR (95% CI)
**Age (for 5-year increase)**	1.41 (1.37, 1.45)***	1.35 (1.30, 1.41)***
**Sex**		
Female	1.00	1.00
Male	1.21 (0.99, 1.48)	0.97 (0.73, 1.30)
**Marital status**		
Married	1.00	1.00
Single	3.19 (2.35, 4.33)**	0.8 (0.55, 1.19)
Widowed	4.44 (2.81, 7.01)**	0.94 (0.57, 1.54)
Divorced	13.81 (9.92, 19.23)**	1.03 (0.69, 1.53)
**Education**		
Unable to read and write	1.00	1.00
Literate	0.25 (0.19, 0.33)***	0.35 (0.13, 0.91)*
**Occupation**		
Unemployed	1.00	1.00
Farmer	13.7 (8.58, 21.82)***	1.3 (0.61, 2.80)
Others	4.14 (2.55, 6.74)***	0.63 (0.30, 1.29)
**Residence**		
Rural	1.00	
Urban	0.85 (0.62, 1.16)	
**Wealth index**		
Poor	1.00	1.00
Medium	1.25 (0.94, 1.65)	0.97 (0.73, 1.31)
Wealthy	0.98 (0.77, 1.26)	0.85 (0.65, 1.11)
Unknown	0.79 (0.53, 1.17)	1.08 (0.72, 1.62)
**Relation to household head**		
Head of household	1.00	1.00
Wife/children	0.17 (0.13, 0.22)***	1.23 (0.82, 1.84)
Extended family & other members	2.30 (1.76, 3.02)***	2.86 (2.05, 3.98)***
**literate#age_5year**		1.18 (1.10, 1.27)***
**Household headship**		
No	1.00	
Yes	3.06 (2.48, 3.77)***	
**Sex of household head**		
Female	1.00	
Male	0.92 (0.71, 1.20)	
**Geographic location**		
Midland	1.00	
Highland	1.12 (0.90, 1.4)	

CI: Confidence interval, HR: Hazard ratio.

*p<0.05.

** p<0.01.

*** p<0.001.

On the other hand, the hazard of mortality from NCDs was not significantly different according to the wealth index of adults. However, it was observed that individuals from medium and wealthy households had a 3% and 15% lower hazard of NCD mortality, respectively, compared to individuals from poor households. Similarly, there was not a statistically significant difference in the mortality hazard from NCD by sex, occupation and marital status (Table 3).

## Discussion

Determining the mortality burden of NCDs was one of the main objectives of this study. We found that, out of the total cause ascertained deaths, NCDs accounted for a higher proportion of death (41.4%) in the predominantly rural community of northern Ethiopia.

The other primary objective of the study was to identify the socio-demographic and economic determinants of NCD mortality. In summary, age, education, relation to head of household and the interaction effect of education and age were the significant determinants of mortality from NCD. In contrast, there was no statistically different NCD mortality by sex, wealth index, occupation and marital status. However, the hazard of NCD mortality decreases with increasing wealth status.

One of our key findings is that the mortality burden from NCDs was slightly higher than infectious causes of death accounting for more than one-third of the total deaths. This clearly indicates that the double mortality burden from both NCDs and communicable diseases is evident in the community. The proportion of mortality burden shared by NCDs, which falls between 31.7% and 37.3% at 95% CI, is higher than in previous studies from Ethiopia [20, 77–79], Africa [80–82] and another cross-continental study conducted in similar research settings located in Africa and Asia [58]. The current finding is also higher than the 30% NCD mortality mentioned in the 2014 WHO mortality report for Ethiopia [83]. However, it is lower than the finding of 56.8% from the Global Burden of Disease (GBD) estimation for 2015 [84]. This study has illuminated the hidden consequences of NCDs, the most severe being mortality. It has uncovered that mortality burden of NCDs has slightly prevailed over communicable diseases or injuries and this substantiates the fact that epi-demographic transition [20, 85–87] is emerging in the community. One of the possible explanations for the slightly higher proportion of NCD-attributed mortality in our population could be due to the significant decline in communicable diseases increasing the life expectancy of Ethiopians. From 1990 to 2015, the age-standardised national annual percentage drop of communicable, neonato-maternal and nutritional diseases was 4.2% [84]. This decrease in communicable diseases could merely be a result of the functional relevance of the investment in public infrastructures such as public health centres particularly during the past decade [88, 89].

The 2015 World Health Statistics release indicated that life expectancy at birth of Ethiopians between 1990s and 2013 increased by about 20 years [15]. Similarly, according to the United Nations Development Report (UNDP), Ethiopia gained a life expectancy of 20.4 years between 1980 and 2014 [88]. Although lower than the above-mentioned results, a more recent global health study by Wang et.al showed that male and female Ethiopians gained a life expectancy of 7.6 and 10.7 years, respectively [90]. Generally, the results show a gain in life expectancy for Ethiopia although there exists variation in the estimated years of gain. Additionally, based on our data, the median age of the deceased individuals was 70 years with an IQR of 59.5–80 years compared to the median age of 31 years with an IQR of 23–46 years for the alive and migrated cohorts. Accordingly, it seems reasonable that we can think of increased life expectancy as one of the partially explaining demographic phenomenon for the comparably high NCD mortality observed in our population [87].

On the other hand, despite Ethiopia’s reportedly significant achievement in economic growth, the country is still one of the poorest nations with a HDI of 0.442, which falls below the average HDI of 0.505 for countries in the low human development group, ranking 174 out of 188 countries [88]. Even though the Federal Ministry of Health (FMoH) has recently started to pay attention and respond to the emergence of NCDs [91], the large burden of communicable diseases, especially among younger age groups, seems to influence much of the operational focus of the ministry office. Moreover, patients’ poor use and access to early diagnosis and treatment, aggravated by the poor health insurance coverage in the study area, may also have contributed to the high NCD attributed mortality. In addition to these factors, the high population-to-health workforce ratio and extremely low density of well-equipped hospitals to population could have exacerbated the mortality from NCDs [15, 41, 88]. Moreover, the healthcare-seeking practice of NCD patients might also be low, as revealed in Malawi [92], eventually leading to premature mortality. Another explanation for this is that only 14.4% of the NCD patients died at health facilities, which can further elucidate the fact that the healthcare services utilization of NCD patients might have been poor assuming death at health facilities, from the context of developing countries, as a proxy indicator of access to and utilization of healthcare services during a terminal illness [41].

In this study, CVDs, cancer and renal failure were the leading NCDs accounting for more than two-thirds, one-fifth and one-tenth of NCD-ascribed deaths respectively. This result is in line with earlier research findings from Ethiopia [20, 77, 79]. The epidemiological fact behind the disproportionate observation of renal failure and ischaemic heart disease attributed deaths among females and oesophageal cancer and chronic liver disease among males is worth investigating. However, our observation of higher occurrence of oesophageal cancer and chronic liver disease as causes of death among males could be related to the higher alcohol consumption among the male population, similar to the study from Sri Lanka [93]. Furthermore, in this study, a large share of the NCD deaths were caused by CVDs. This is consistent with other studies which demonstrated that CVDs are the single most important cause of mortality and disability in low and middle-income countries [14, 18, 80, 94–97].

In this study, a 5-year increase in age was associated with 35% higher hazard of mortality from NCDs. In 2015, aging was reported to be the most important factor for the increased occurrence of NCD morbidity and mortality [7]. This might be justified by the fact that NCD risk factors are likely to accumulate with increasing age [58, 98].

Our study provides strong insight into the inverse effect of education on NCD-attributed mortality. Adjusting the effects of the fitted covariates, adults who were unable to read and write were at a higher hazard of mortality from NCDs compared to those who were literate. Our finding substantiates several previous prospective study findings which demonstrated that lower educational attainment is associated with higher cardiovascular, cancer and other NCD-related mortality [31–33, 38, 99–101]. The significant effect of education on reducing NCD attributed mortality may partly be explained by lower healthcare utilisation and disparity in the severity of disease prognosis among the non-literate group [33, 41]. Moreover, education could promote the adoption of healthy behaviours more easily and the avoidance of unhealthy habits which could finally avert mortality from NCDs such as diabetes mellitus and enhance healthcare-seeking behaviour during terminal illness [32, 102]. Although our observation of a strong association of literacy with reduction of NCD mortality is not adjusted for the established lifestyle NCD risk factors, in two largest prospective cohort studies, that had 303,036 and 39, 228 study participants, it was reported that lower education remained a significant predictor of mortality from CVDs or premature mortality, even after adjustment for these factors [31, 33]. This finding may have an important public health implication with respect to preventing adult mortality from NCDs. The possibly higher health service utilization by the literate group, as shown in previous studies, could partly account for the observed difference among the cohorts [41]. However, further research needs to be undertaken to decode the details of different pathways on how education could mediate or causally influence NCD mortality in the rural setting of the population. This could be of paramount importance for designing effective interventions to address NCD mortality. In this study, the protective effect of literacy was strongly modified by increasing age. This significant attenuating effect of age on the protective effect of education may highlight the reality that aging stands as a dominant risk factor to NCD-attributed mortality.

One of the interesting findings of our study was that NCD-attributed mortality was significantly different depending on the type of relationship household members have with their respective head of household. Extended family and non-family household members were at a significantly increased hazard of NCD mortality compared to the household head. As to our update, published research works linking adult mortality from NCDs and household headship are unavailable. Yet, one study found that being head of household significantly reduced elderly mortality for both sexes. Nonetheless, this effect was found to decline with increasing age of the household heads [57]. The relationship of family members to the head of households has also a vital implication to the survival of children. There is supporting evidence which shows that children who lived in households headed by persons other than their parents faced a relative survival disadvantage [56]. It has been reported that NCDs pose a significant economic burden to households by means of catastrophic hospitalization expenditure and productivity loss [9, 10]. In developing countries, where the health care costs are mostly covered by the patients, the adverse economic effect of catastrophic out of pocket expenses disproportionately affects the poor and vulnerable segments of the population [10]. As such, it is possible that the healthcare-seeking practice and access to medicines by these vulnerable household members might have been excessively impeded which could have led to the significantly excess hazard of NCD ascribed mortality among these co-residents. Moreover, extended family and non-family members are less likely to access household resources since, in the cultural context of the study area, household related decisions, such as spending decisions, are primarily made by the heads of the households. Similarly, in one longitudinal study, it was explained that joint household resources were allocated differentially and accessed greatly by household heads and their wives [57]. Furthermore, illness from NCDs is generally characterised by long-term morbidity compared to communicable diseases, which could also predispose NCD patients who were not closely related to the household heads, to poorer care-seeking behaviour. This could evidently be aggravated by lifelong medical expenditures and the probable family fatigue as a result of long-term care [10, 103]. Conclusively, we observed that intra-household structure and relationships have significance in terms of excess NCD-related mortality among extended family and non-family co-residents within households. Therefore, we strongly recommend that public health interventions, such as community-based health insurance, should consider the intra-household structure and relationships, particularly giving focus to vulnerable co-residents, to significantly prevent NCD-attributed mortality.

Our study did not find a statistically significant difference in NCD mortality by wealth index. Nevertheless, the study demonstrated that individuals who had wealthy asset status had a lower hazard of NCD mortality compared to those who were poor individuals. This may show the positive survival gain of higher economic status in relation to reducing the hazard of adult mortality from NCDs. Similar to our study, other groups did not find a statistically significant difference between wealth and acute myocardial infarction mortality (AMI) [104], wealth and cancer mortality [55]. In contrast, several other studies showed a statistically significant association of wealth with all-cause and NCD mortality [52, 53, 55, 105]. The reason for the finding of insignificant association of wealth with NCD mortality is unclear, but it could be partially attributed to the fact that non-governmental micro-lending service is commonly practiced in the study area, which could have diluted the effect of wealth status on NCD mortality. On the other hand, for some reasons, it is mentioned that wealth status of individuals estimated from household wealth measures, such as household wealth index, could be an unreliable measure of individual economic position [106]. Controlling the effect of education and all other fitted covariates, unemployment was not a significant factor for NCD mortality. This finding is rather contradictory to the evidence from previous research works [44, 46]. This could be explained by the strong correlation of education, income and occupation; however, education is consistently the strongest indicator, proofing its causal effect, compared to the two parameters [27–30].

The results of this study could be generalizable to many developing countries taking in to account the mostly similar situations of low literacy level and patricentric household headship, which is a proxy indicator of differential individual access to within household resources [57], in most rural areas of such countries. One should, however, notice that the results should be utilized with great prudence because NCD patients living in these different countries are exposed to different health system, social and cultural contexts, where the generalization may not be applicable. Contrary to this, we expect that our findings may not be generalized to NCD patients living in developed countries due to the likely better survival resulting from better health insurance coverage. Moreover, the higher literacy level and different family composition and structure in developed countries may also create distinctive contexts where the results of the current study may not be directly inferred. Nevertheless, this does not imply that the results will not have relevance to such economically thrived part of the world.

Our study is based on highly trained and experienced enumerators who collected the population-based longitudinal data using standardized data collection tools and procedures. An additional strength of the study is that it is based on a large number of participants followed over long period of time. However, the study is not without limitations. One major limitation of the study is that undetermined and unspecified causes of death, 16.8% and 3.6% respectively, accounted for about one-fifth of the total causes of death. This may be due to the fact that physician review based verbal autopsy is less accurate in determining causes of death as compared to other methods [65, 107]. It could be difficult to clearly determine how this has already impacted our results; however, assuming aging as a principal factor for the occurrence of NCDs among elderly adults [98], we think that this might have reduced the burden of NCD mortality because 76.6% of the undetermined and unspecific causes were among adults aged 65 years and above. The second limitation is that the absence of data for some important variables such as the classical NCD risk factors, which may further explain the disparity in NCD mortality, should also be mentioned as a limitation of our study.

## Conclusion

In summary, our study has found a high mortality burden from NCDs. The evidence from this study indicates that older age, non-literacy, and being an extended family or non-family member of the household head, show a disparity of significantly higher NCD attributed adult mortality in the project area. This study has enhanced our understanding of social determinants in the context of a predominantly rural community of Ethiopia. Future public health interventions should integrate these findings to plan interventions in those vulnerable groups to meaningfully reduce adult NCD mortality. Further research, combining quantitative and qualitative approaches, needs to be implemented to disentangle the different possible pathways education and intra-household composition and relationships follow to result in differential adult mortality from NCDs.

## Supporting information

S1 Fig A conceptual framework of the social determinants of NCD attributed adult mortality. (TIF)

S1 Table Predictors of NCD mortality based on sensitivity analysis. (RTF)

S1 Data Collection tools (ZIP)
