# Supplementary figures and images for: Social determinants of adult mortality from non-communicable diseases in northern Ethiopia, 2009-2015: Evidence from health and demographic surveillance site
Source: PLoS One. 2017 Dec 13;12(12):e0188968. doi: 10.1371/journal.pone.0188968 (PMC5728486; doi:10.1371/journal.pone.0188968)

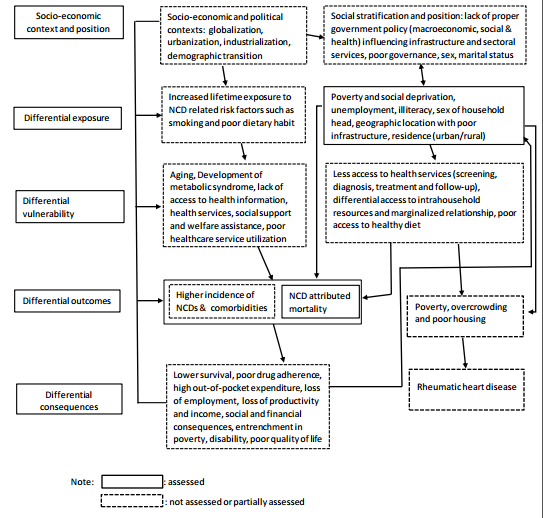

Supplement: S1 Fig — (TIF) [file pone.0188968.s001.tif]
