# Supplementary material for: Social determinants of adult mortality from non-communicable diseases in northern Ethiopia, 2009-2015: Evidence from health and demographic surveillance site
Source: PLoS One. 2017 Dec 13;12(12):e0188968. doi: 10.1371/journal.pone.0188968 (PMC5728486; doi:10.1371/journal.pone.0188968)
Supplement: S1 Table — (RTF) [file pone.0188968.s002.rtf]

S 1 Table. Predictors of NCD mortality based on sensitivity analysis
Variables	Adjusted HR (95% CI)	
Age (for 5 year increase )	1.35 (1.29, 1.40)***	
Sex		
  Female	1.00	
  Male	0.99(0.74, 1.32)	
Marital status		
  Married	1.00	
  Single	0.73(0.49, 1. 07)	
  Widowed	0.90 (0.55, 1.48)	
  Divorced	0.96 (0.65, 1.41)	
Education		
  Unable to read and write	1.00	
  Literate	0.48 (0.19, 1.26)	
Occupation		
  Unemployed	1.00	
  Farmer	1.14 (0.53, 2.43)	
  Others	0.58 (0.28, 1.20)	
Wealth index		
  Poor	1.00	
  Medium	0.94 (0.70, 1.26)	
  Wealthy	0.82 (0.62, 1.07)	
  Unknown	1.02 (0.68, 1.54)	
Relation to household head		
  Head of household	1.00	
  Wife/children	1.20 (0.80, 1.81)	
  Extended family & other members	2.90 (2.08, 4.05)***	
literate#age_5year	1.16 (1.07, 1.24)***	


CI:Confidence interval, HR: Hazard ratio, *p<0.05, ** p<0.01 and *** p<0.001
