# Supplementary material for: Social determinants of adult mortality from non-communicable diseases in northern Ethiopia, 2009-2015: Evidence from health and demographic surveillance site
Source: PLoS One. 2017 Dec 13;12(12):e0188968. doi: 10.1371/journal.pone.0188968 (PMC5728486; doi:10.1371/journal.pone.0188968)
Supplement: S1 Data Collection tools — (ZIP) [file pone.0188968.s003.zip › S2 data collection tools/VA Questionnaire 3 English.doc]

**VERBAL AUTOPSY QUESTIONNAIRE 3:**

**DEATH OF A PERSON AGED 15 YEARS AND ABOVE**

| **CONSENT STATEMENT**  Good Morning/Good Afternoon/ Good Evening.  My name is ____________________________and I am working with Jimma University.  We are collecting information on the causes of death in the community. We would very much appreciate your participation in this effort. We want to ask you about the circumstances leading to the death of the deceased. Whatever information you provide will be kept strictly confidential. No information identifying you or the deceased will ever be released to anyone outside of this information-collection activity. Participation in this survey is voluntary and you can choose not to answer any individual question or all of the questions. You may also stop the interview completely at any time without any consequences at all. However, we hope that you will participate in this survey since the results will help the government improve services for people.  At this time, do you want to ask me anything about the purpose or content of this interview?  May I begin the interview now?  Signature of interviewer: ______________________________ Date: ________________________  RESPONDENT AGREES TO BE INTERVIEWED ….. 1 Respondent does not agree to be interviewed …… 2 🡲 END **🢃** | | | | | | |
| --- | --- | --- | --- | --- | --- | --- |
| **Section 1: VA Interviewer Contact Result** | | | | | | |
| Name of interviewer: ______________________ | | | Kebele name __________________  Gote _______________________ | | | Family ID  ___________ |
| Interviewer signature : ______________________ | | |  |  |  |  |
| Name of supervisors: ______________________ | | | Gere _______________________ | | | |
| Supervisors signature : ______________________ | | | House number: ______________________ | | | |
| Outcome of first visit | 1.Completed 2. Not around home 3. Interrupted 4. Refused | | |  | Date of appointment for other time visit ______________________ | |
| Outcome of second visit | 1.Completed 2. Not around home 3. Interrupted 4. Refused | | |  | Date of appointment for other time visit ______________________ | |
| Outcome of third Visit | 1.Completed 2. Not around home 3. Interrupted 4. Refused | | |  | Date of appointment for other time visit ______________________ | |
| Date of interview (ETC): Day/Month/Year | | ______/______/________ | | | | |

| **SECTION 2. BASIC INFORMATION ABOUT RESPONDENT** | | | |
| --- | --- | --- | --- |
| 201 | RECORD THE TIME AT START OF INTERVIEW | HOUR ……………………….  MINUTES ………………….. |  |
| 202 | NAME OF THE RESPONDENT | _____________________________  (NAME) |  |
| 203 | Sex of respondent | Female …………………………….. 1  Male ………………………………… 2 |  |
| 204 | Age of respondent | In Years |  |
| 205 | What is your relationship to the deceased? | FATHER/ MOTHER …………………1  SPOUSE…………………………. …. 2  SIBLING ………………..……………3  OTHER RELATIVE _______________ 4  (SPECIFY)  NO RELATION ……………………… 5 |  |
| 206 | For how long have you lived with the deceased in the period leading to her/his death? | From the first day of illness…………1  After the start of the illness ………,..2  Few days before her/his death …… 3 |  |

| **SECTION 3. INFORMATION ON THE DECEASED AND DATE/PLACE OF DEATH** | | | |
| --- | --- | --- | --- |
| 301 | What was the name of the deceased? | _____________________________  (NAME) |  |
| 302 | Was the deceased female or male? | FEMALE ………………………………1  MALE ………………………………….2 |  |
| 303 | When was the deceased born?  RECORD ‘9 8’ IF DON’T KNOW DAY OR MONTH RECORD ‘9 9 9 8’ IF DON’T KNOW YEAR | DAY ………………………  MONTH ………………….  YEAR …………………….. |  |
| 304 | How old was the deceased when s/he died? | AGE IN YEARS…………………… |  |
| 305 | When did s/he die?  RECORD ‘98’ IF DON’T KNOW DAY OR MONTH RECORD ‘9998’ IF DON’T KNOW YEAR | Day …………………………..  Month …………………………  Year …………………….. |  |
| 306 | Where did s/he die? | Hospital ………………………………….1  Health center…………………………….2  Other health faculty………....................3  Home……………………………………..4  Other __________________________5  (specify)  Don’t know……………………………….8 |  |
| 307 | For deaths at hospital or health facility, record facility name and address: ______________________________________________________________________ ______________________________________________________________________ | |  |
| 308 | What was her/his occupation, that is what kind or work did s/he mainly do? | Farmer ………….................. 1  Student … …………………. 2  Merchant ………….……… 3  Shepard ……….………….. 4  Daily laborer/house maid … 5  Housewife …………….……. 6  Government employee ………7  Unemployed …………..……. 8  Other (specify)……………… 9  Don’t know ………………………..98 |  |
| 309 | What was the highest level of formal education the deceased attended? | Illiterate ………….................. 1  Grade 1 – 4 …………………. 2  Grade 5 – 8 ………………… 3  Grade 9 – 10 ……………….. 4  Grade 11 – 12 ……………… 5  Don’t know ………………… 8 |  |
| 310 | What was her/his marital status? | Never married …………………………..1  Married/living with a partner …………. 2  Cohabitation ……………………………3  Widowed ……………………………….4  Divorced ……………………………….5  Separated ………………………………6  Don’t know ……………………………8 |  |
|  |  |  |  |

| **SECTION 4. RESPONDENT’S ACCOUNT OF ILLNESS/EVENTS LEADING TO DEATH** | | |
| --- | --- | --- |
| 401 | Could You Tell Me About the Illness/Events that Led To Her/His Death?  **Note: When possible, use local term for the symptom**.  _______________________________________________________________________  ___________________________________________________________________  _______________________________________________________________________  ___________________________________________________________________  _______________________________________________________________________  ___________________________________________________________________  _______________________________________________________________________  ___________________________________________________________________  _______________________________________________________________________  ___________________________________________________________________  _______________________________________________________________________  ___________________________________________________________________  _______________________________________________________________________  _______________________________________________________________________ |  |
| 402 | CAUSE OF DEATH 1 ACCORDING TO RESPONDENT  _________________________________________________________ |  |
| 403 | CAUSE OF DEATH 2 ACCORDING TO RESPONDENT  _________________________________________________________ |  |

| **SECTION 5. HISTORY OF PREVIOUSLY KNOWN MEDICAL CONDITIONS** | | | |
| --- | --- | --- | --- |
| I would like to ask you some questions concerning previously known medical conditions the deceased had; injuries and accidents that the deceased suffered; and signs and symptoms that the deceased had/showed when s/he was ill. Some of these questions may not appear to be directly related to his/her death. Please bear with me and answer all the questions. They will help us to get a clear picture of all possible symptoms that the deceased had.  Please tell me if the deceased suffer from any of the following illnesses: | | | |
| 501 | High blood pressure | Yes ………………………………………1  No ………………………………………..2  Don’t know ………………………………8 |  |
| 502 | Diabetes? | Yes ………………………………………1  No ………………………………………..2  Don’t know ………………………………8 |  |
| 503 | Asthma | Yes ………………………………………1  No ………………………………………..2  Don’t know ………………………………8 |  |
| 504 | Epilepsy? | Yes ………………………………………1  No ………………………………………..2  Don’t know ………………………………8 |  |
| 505 | Malnutrition? | Yes ………………………………………1  No ………………………………………..2  Don’t know ………………………………8 |  |
| 506 | Cancer? | Yes ………………………………………1  No ………………………………………..2  Don’t know ………………………………8 | —**►508**  —**►508** |
| 507 | Can you specify the type or site of cancer? | Type/site ___________________________  ___________________________________ |  |
| 508 | Tuberculosis? | Yes ………………………………………1  No ………………………………………..2  Don’t know ………………………………8 |  |
| 509 | HIV/AIDS? | Yes ………………………………………1  No ………………………………………..2  Don’t know ………………………………8 |  |
| 510 | Did s/he suffer from any other medically diagnosed illness? | Yes ………………………………………1  No ………………………………………..2  Don’t know ………………………………8 | —**►601**  —**►601** |
| 511 | Can you specify the illness? | Illness _____________________________  ___________________________________ |  |

| **SECTION 6. HISOTRY OF INJURIES/ACCIDENTS** | | | |
| --- | --- | --- | --- |
| 601 | Did s/he suffer from any injury or accident that led to her/his death? | Yes ……………………………………1  No ……………………………………..2  Don’t know ……………………………8 | —**►604**  —**►604** |
| 602 | What kind of injury or accident did the deceased suffer? | Road traffic accident ……………… 01 Fall ………………………………… 02 Drowning …………………………. 03 Poisoning ……………………….. … 04 Burns ………………………………. 05 Violence/assault ………………..... 06 Other ________________________96  (specify)  Don’t know ………………………98 |  |
| 603 | Was the injury or accident intentionally inflicted by someone else? | Yes ………………………………….….1  No …………………………………….. 2  Don’t know …………………………… 8 | —**►607** |
| 604 | Do you think that s/he committed suicide? | Yes ………………………………….….1  No …………………………………….. 2  Don’t know …………………………… 8 |  |
| 605 | Did s/he suffer from any animal/insect bite that led to her/his death? | Yes ………………………………….….1  No …………………………………….. 2  Don’t know …………………………… 8 | —**►607**  —**►607** |
| 606 | What type of animal/insect? | Dog …………………………………….1  Snake ………………………………….2  Insect …………………………………..3  Other ________________________ 6  (Specify)  Don’t know ……………………………8 |  |
| 607 | CHECK QUESTION 302 FOR SEX AT DEATH:    FEMALE MALE  701 | | 901 |

| **SECTION 7. SYMPTOMOS AND SIGNS ASSOCIATED WITH ILLNESS OF WOMEN** | | | |
| --- | --- | --- | --- |
| 701 | Did she have an ulcer or swelling in the breast? | Yes ………………………………….….1  No …………………………………….. 2  Don’t know …………………………… 8 | —**►703**  —**►703** |
| 702 | For how long did she have an ulcer or swelling in the breast? | Days ……………………….  Months ………………………..  Don’t know ……………............9 9 8 |  |
| 703 | Did she have excessive vaginal bleeding during menstrual periods? | Yes ………………………………….….1  No …………………………………….. 2  Don’t know …………………………… 8 | —**►705**  —**►705** |
| 704 | For how long did s/he have the excessive vaginal bleeding during menstrual periods? | Days ……………………….  Months ………………………..  Don’t know ……………............98 |  |
| 705 | Did she have vaginal bleeding in between menstrual periods? | Yes ………………………………….….1  No …………………………………….. 2  Don’t know …………………………… 8 | —**►707**  —**►707** |
| 706 | For how long did she have vaginal bleeding in between menstrual periods? | Days ……………………….  Months ………………………..  Don’t know ……………............98 |  |
| 707 | Did she have abnormal vaginal discharge? | Yes ………………………………….….1  No …………………………………….. 2  Don’t know …………………………… 8 | —**►801**  —**►801** |
| 708 | For how long did she have abnormal vaginal discharge? | DAYS ………………………….  Months ………………………..  Don’t know ……………............98 |  |

| **SECTION 8. SYMPTOMS AND SIGNS ASSOCIATED WITH PREGNANCY** | | | | | | |
| --- | --- | --- | --- | --- | --- | --- |
| 801 | | Was she pregnant at the time of death | | Yes ………………………………….….1  No …………………………………….. 2  Don’t know …………………………… 8 | —**►806**  —**►806** | |
| 802 | | How long was she pregnant? | | weeks ………………………….  Months ………………………..  Don’t know ……………............98 |  | |
| 803 | | How many pregnancies had she had, including this one? | | number………………………….  Don’t know …………………… 8 |  | |
| 804 | During the last 3 months of pregnancy, did she suffer from any of the following illnesses:   1 Vaginal bleeding? 2 Smelly vaginal discharge? 3 Puffy face? 4 Headache? 5 Blurred vision? 6 Convulsion? 7 Febrile illness? 8 Severe abdominal pain that was not labor pain? 9 Pallor and shortness of breath (both present)? 10 Did she suffer from any other illness? | | **YES NO DK if yes at**  **what month**  Vaginal bleeding…….. 1 2 8 _______  Smelly vaginal discharge 1 2 8 ______  Puffy face ………………. 1 2 8_______  Headache ……………. 1 2 8 _______ Blurred vision ………….. 1 2 8_______  Convulsion …………… 1 2 8 _______ Febrile illness …… …. 1 2 8 _______  severe abdominal pain  (not labor pain) …….. 1 2 8________  Pallor/shortness  of breath (both)…………1 2 8_______  other illness ………………1 2 8_______   specify______________________ | | |  |
| 805 | Did she die during labor, but undelivered? | | Yes ………………………………….….1  No …………………………………….. 2  Don’t know …………………………… 8 | | |  |

| 806 | Did she give birth recently? | Yes ………………………………….….1  No …………………………………….. 2  Don’t know …………………………… 8 | —**►817**  —**►817** |
| --- | --- | --- | --- |
| 807 | How many days after giving birth did she die? | DAYS ………………………….  Months ………………………..  Don’t know ……………............98 |  |
| 808 | Was there excessive bleeding during labor before delivering the baby? | Yes ………………………………….….1  No …………………………………….. 2  Don’t know …………………………… 8 |  |
| 809 | Was there excessive bleeding after delivering the baby? | Yes ………………………………….….1  No …………………………………….. 2  Don’t know …………………………… 8 |  |
| 810 | Did she have difficulty in delivering the placenta? | Yes ………………………………….….1  No …………………………………….. 2  Don’t know …………………………… 8 |  |
| 811 | Was she in labor for unusually long (more than 12 hours)? | Yes ………………………………….….1  No …………………………………….. 2  Don’t know …………………………… 8 |  |
| 812 | Was it a normal vaginal delivery? | Yes ………………………………….….1  No …………………………………….. 2  Don’t know …………………………… 8 | —**►814**  —**►814** |

| 813 | What type of delivery was it? | | Forceps/Vacuum………………………1  Caesarean section ……………………2  Other _________________________6  (Specify)  Don’t know …………………………….8 | |  | |
| --- | --- | --- | --- | --- | --- | --- |
| 814 | Did she have foul smelling vaginal discharge? | | Yes ………………………………….….1  No …………………………………….. 2  Don’t know …………………………… 8 | |  | |
| 815 | Where did she give birth? | | Hospital ………………………………..1  Health center………………………….2  Clinic……………..……........................3  Health post……………………………..4  Home…………………………………...5  Other ________________________ 6  (specify)  Don’t know…………………………….8 | |  | |
| 816 | Who conducted the delivery? | | Doctor ………………………………….1  Nurse/Midwife………………………….2  Traditional birth attendant ……………3  Relative ………………………………..4  Mother by herself ……………………. 5  Other __________________________  (Specify)  Don’t know …………………………….8 | |  | |
| 817 | Did she experience an abortion recently? | | Yes ………………………………….….1  No …………………………………….. 2  Don’t know …………………………… 8 | | —**►901**  —**►901** | |
| 818 | Did she die during the abortion? | | Yes ………………………………….….1  No …………………………………….. 2  Don’t know …………………………… 8 | | —**►820**  —**►820** | |
| 819 | How many days before death did she have the abortion? | | Days ………………………….  Don’t know …………………… 98 | |  | |
| 820 | How many months pregnant was she when she had the abortion? | | Months ……………………….  Don’t know …………………… 98 | |  | |
| 821 | | Did she have heavy bleeding after the abortion? | | Yes ………………………………….….1  No …………………………………….. 2  Don’t know …………………………… 8 | |  |
| 822 | | Did the abortion occur by itself, spontaneously? | | Yes ………………………………….….1  No …………………………………….. 2  Don’t know …………………………… 8 | | —**►901**  —**►901** |
| 823 | | Did she take medicine or treatment to induce? | | Yes ………………………………….….1  No …………………………………….. 2  Don’t know …………………………… 8 | |  |

| **SECTION 9. SIGNS AND SYMPTOMS NOTED DURING THE FINAL ILLNESS** | | | |
| --- | --- | --- | --- |
| 901 | For how long was s/he ill before s/he died? | DAYS ………………………….  Months ………………………..  Don’t know ……………............98 |  |
| 902 | Did s/he have a fever? | Yes ………………………………….….1  No …………………………………….. 2  Don’t know …………………………… 8 | —**►906**  —**►906** |
| 903 | For how long did s/he have a fever? | Days……………………………  Months ………………………..  Don’t know ……………............98 |  |
| 904 | Was the fever continuous or on and off? | Continuous ……………………………1  On and Off …………………………….2  Don’t know ……………………………8 |  |
| 905 | Did s/he have fever only at night? | Yes ………………………………….….1  No …………………………………….. 2  Don’t know …………………………… 8 |  |
| 906 | Did s/he have chills/rigor? | Yes ………………………………….….1  No …………………………………….. 2  Don’t know …………………………… 8 |  |
| 907 | Did s/he have a cough? | Yes ………………………………….….1  No …………………………………….. 2  Don’t know …………………………… 8 | —**►913**  —**►913** |
| 908 | For how long did s/he have a cough? | DAYS ………………………….  Months ………………………..  Don’t know ……………............998 |  |
| 909 | Was the cough severe? | Yes ………………………………….….1  No …………………………………….. 2  Don’t know …………………………… 8 |  |
| 910 | Was the cough productive with sputum? | Yes ………………………………….….1  No …………………………………….. 2  Don’t know …………………………… 8 |  |
| 911 | Did s/he cough out blood? | Yes ………………………………….….1  No …………………………………….. 2  Don’t know …………………………… 8 |  |
| 912 | Did s/he have night sweats? | Yes ………………………………….….1  No …………………………………….. 2  Don’t know …………………………… 8 |  |
| 913 | Did s/he have breathlessness? | Yes ………………………………….….1  No …………………………………….. 2  Don’t know …………………………… 8 | —**►918**  —**►918** |
| 914 | For how long did s/he have breathlessness? | DAYS ………………………….  Months ………………………..  Don’t know ……………............998 |  |
| 915 | Was s/he unable to carry out daily routines due to breathlessness? | Yes ………………………………….….1  No …………………………………….. 2  Don’t know …………………………… 8 |  |
| 916 | Was s/he breathless while lying flat? | Yes ………………………………….….1  No …………………………………….. 2  Don’t know …………………………… 8 |  |
| 917 | Did s/he have wheezing? | Yes ………………………………….….1  No …………………………………….. 2  Don’t know …………………………… 8 |  |
| 918 | Did s/he have chest pain? | Yes ………………………………….….1  No …………………………………….. 2  Don’t know …………………………… 8 | —**►928**  —**►928** |
| 919 | For how long did s/he have chest pain? | DAYS ………………………….  Months ………………………..  Don’t know ……………............998 |  |
| 920 | Did chest pain start suddenly or gradually? | Suddenly……………. …………….…. 1  Gradually ………………………………2  Don’t know …………………………… 8 |  |
| 921 | When s/he had severe chest pain, how long did it last? | Less than half an hour ……………….1  Half an hour to 24 hours ……………..2  Longer than 24 hours ………………...3  Don’t know …………………………….8 |  |
| 922 | Was the chest pain located below the breastbone (sternum)? | Yes ………………………………….….1  No …………………………………….. 2  Don’t know …………………………… 8 |  |
| 923 | Was the chest pain located over the heart and did it spread to the left arm? | Yes ………………………………….….1  No …………………………………….. 2  Don’t know …………………………… 8 |  |
| 924 | Was the chest pain located over the ribs (sides)? | Yes ………………………………….….1  No …………………………………….. 2  Don’t know …………………………… 8 |  |
| 925 | Was the chest pain continuous or on and off? | Continuous ……………………………1  On and Off …………………………….2  Don’t know ……………………………8 |  |
| 926 | Did the chest pain get worse while coughing? | Yes ………………………………….….1  No …………………………………….. 2  Don’t know …………………………… 8 |  |
| 927 | Did s/he have palpitations? | Yes ………………………………….….1  No …………………………………….. 2  Don’t know …………………………… 8 |  |
| 928 | Did s/he have diarrhea? | Yes ………………………………….….1  No …………………………………….. 2  Don’t know …………………………… 8 | —**►933**  —**►933** |
| 929 | For how long did s/he have diarrhea? | Days…………………….  Months ………………………..  Don’t know ……………............998 |  |
| 930 | Was the diarrhea continuous or on and off? | Continuous ……………………………1  On and Off …………………………….2  Don’t know ……………………………8 |  |
| 931 | At any time during the final illness was there blood in the stool? | Yes ………………………………….….1  No …………………………………….. 2  Don’t know …………………………… 8 |  |
| 932 | When the diarrhea was most severe, how many times did s/he pass stools in a day? | Number……………………….  Don’t know …………………… 9 8 |  |

| 933 | Did s/he vomit? | Yes ………………………………….….1  No …………………………………….. 2  Don’t know …………………………… 8 | —**►937**  —**►937** |
| --- | --- | --- | --- |
| 934 | For how long did s/he vomit? | Days…………………….  Months ………………………..  Don’t know ……………............9 9 8 |  |
| 935 | Did the vomit look like a coffee-colored fluid or bright red/blood red or some other? | Coffee-colored fluid …………………..1  Bright read/blood red ………………...2  Other ________________________ 6  (Specify)  Don’t know …………………………….8 |  |
| 936 | When the vomiting was most severe, how many times did s/he vomit in a day? | Number……………………….  Don’t know …………………… 9 8 |  |
| 937 | CHECK QUESTION 302 SEX OF THE DECEASED:  FEMALE MALE | | **939** |
| 938 | CHECK QUESTION 801, 805, 819, TO SEE IF SHE DIED DURING  PREGNANCY, LABOR, ABORTION OR POSTPARTUM:  NO YES | | **948** |
| 939 | Did s/he have abdominal pain? | Yes ………………………………….….1  No …………………………………….. 2  Don’t know …………………………… 8 | —**►941**  —**►941** |
| 940 | For how long did s/he have abdominal pain? | Days…………………….  Months ………………………..  Don’t know ……………............98 |  |
| 941 | Did s/he have abdominal distension? | Yes ………………………………….….1  No …………………………………….. 2  Don’t know …………………………… 8 | —**►945**  —**►945** |
| 942 | For how long did s/he have abdominal distension? | Days…………………….  Months ………………………..  Don’t know ……………............98 |  |
| 943 | Did the distension develop rapidly within days or gradually over months? | Rapidly within days …………………1  Gradually over months ……………..2  Don’t know …………………………..8 |  |
| 944 | Was there a period of a day or longer during which s/he did not pass any stool? | Yes ………………………………….….1  No …………………………………….. 2  Don’t know …………………………… 8 |  |
| 945 | Did s/he have any mass in the abdomen? | Yes ………………………………….….1  No …………………………………….. 2  Don’t know …………………………… 8 | —**►948**  —**►948** |
| 946 | For how long did s/he have the mass in the abdomen? | Days…………………….  Months ………………………..  Don’t know ……………............998 |  |
| 947 | Where in the abdomen was the mass located? | Right upper abdomen ……………….1  Left upper abdomen …………………2  Lower abdomen ……………………...3  All over abdomen …………………….4  Don’t know ……………………………8 |  |
| 948 | Did s/he have difficulty or pain while swallowing solids? | Yes ………………………………….….1  No …………………………………….. 2  Don’t know …………………………… 8 | —**►950**  —**►950** |
| 949 | For how long did s/he have difficulty or pain while swallowing solids? | Days……………………………  Months ………………………..  Don’t know ……………............98 |  |
| 950 | Did s/he have difficulty or pain while swallowing liquids? | Yes ………………………………….….1  No …………………………………….. 2  Don’t know …………………………… 8 | —**►952**  —**►952** |
| 951 | For how long did s/he have difficulty or pain while swallowing liquids? | Days…………………….  Months ………………………..  Don’t know ……………............98 |  |
| 952 | Did s/he have headache? | Yes ………………………………….….1  No …………………………………….. 2  Don’t know …………………………… 8 | —**►955**  —**►955** |
| 953 | For how long did s/he the have headache? | Days…………………….  Months ………………………..  Don’t know ……………............998 |  |
| 954 | Was the headache severe? | Yes ………………………………….….1  No …………………………………….. 2  Don’t know …………………………… 8 |  |
| 955 | Did s/he have a stiff or painful neck? | Yes ………………………………….….1  No …………………………………….. 2  Don’t know …………………………… 8 | —**►957**  —**►957** |
| 956 | For how long did s/he have a stiff or painful neck? | Days ………………………….  Don’t know …………………… 98 |  |
| 957 | Did s/he have mental confusion? | Yes ………………………………….….1  No …………………………………….. 2  Don’t know …………………………… 8 | —**►960**  —**►960** |
| 958 | For how long did s/he have mental confusion? | Days…………………….  Months ………………………..  Don’t know ……………............98 |  |

| 959 | Did the mental confusion start suddenly, quickly within a single day, or slowly over many days? | Suddenly……………. …………….…. 1  Within a day (Fast)……………………2  Slowly (Many days)…………………...3  Don’t know …………………………… 8 |  |  |
| --- | --- | --- | --- | --- |
| 960 | Did s/he become unconscious? | Yes ………………………………….….1  No …………………………………….. 2  Don’t know …………………………… 8 | —**►963**  —**►963** |  |
| 961 | For how long was s/he unconscious? | Days…………………….  Months ………………………..  Don’t know ……………............98 |  |  |
| 962 | Did the unconsciousness start suddenly, quickly within a single day, or slowly over many days? | Suddenly……………. …………….…. 1  Within a day (Fast)……………………2  Slowly (Many days)…………………...3  Don’t know …………………………… 8 |  |  |
| 963 | Did s/he have convulsions? | Yes ………………………………….….1  No …………………………………….. 2  Don’t know …………………………… 8 | —**►965**  —**►965** | |
| 964 | For how long did s/he have convulsions? | Days…………………….  Months ………………………..  Don’t know ……………............98 |  | |
| 964.1 | How many times did s/he have the convulsion during the illness? | Number……………………………..  Don’t know …………………………98 |  | |
| 965 | Was s/he unable to open the mouth? | Yes ………………………………….….1  No …………………………………….. 2  Don’t know …………………………… 8 | —**►967**  —**►967** | |
| 966 | For how long was s/he unable to open the mouth? | Days ………………………….  Don’t know …………………… 98 |  | |
| 967 | Did s/he have stiffness of the whole body? | Yes ………………………………….….1  No …………………………………….. 2  Don’t know …………………………… 8 | —**►969**  —**►969** | |
| 968 | For how long did s/he have stiffness of the whole body? | Days ………………………….  Don’t know …………………… 98 |  | |
| 969 | Did s/he have paralysis of one side of the body? | Yes ………………………………….….1  No …………………………………….. 2  Don’t know …………………………… 8 | —**►972**  —**►972** | |
| 970 | For how long did s/he have paralysis of one side of the body? | Days…………………….  Months ………………………..  Don’t know ……………............98 |  | |
| 971 | Did the paralysis of one side of the body start suddenly, quickly within a single day, or slowly over many days? | Suddenly……………. …………….…. 1  Within a day (Fast)……………………2  Slowly (Many days)…………………...3  Don’t know …………………………… 8 |  | |
| 972 | Did s/he have paralysis of the lower limbs? | Yes ………………………………….….1  No …………………………………….. 2  Don’t know …………………………… 8 | —**►975**  —**►975** | |

| 973 | How long did s/he have paralysis of the lower limbs? | Days………………………….  Months ………………………..  Don’t know ……………............98 |  |
| --- | --- | --- | --- |
| 974 | Did the paralysis of the lower limbs start suddenly, quickly within a single day, or slowly over many days? | Suddenly……………. …………….…. 1  Within a day (Fast)……………………2  Slowly (Many days)…………………...3  Don’t know …………………………… 8 |  |
| 975 | Was there any change in color of urine? | Yes ………………………………….….1  No …………………………………….. 2  Don’t know …………………………… 8 | —**►977**  —**►977** |
| 976 | For how long did s/he have the change in color of urine? | Days ………………………….  Months ………………………..  Don’t know ……………............98 |  |
| 977 | During the final illness did s/he ever pass blood in the urine? | Yes ………………………………….….1  No …………………………………….. 2  Don’t know …………………………… 8 | —**►979**  —**►979** |
| 978 | For how long did s/he pass blood in the urine? | Days ………………………….  Months ………………………..  Don’t know ……………............998 |  |
| 979 | Was there any change in the amount of urine s/he passed daily? | Yes ………………………………….….1  No …………………………………….. 2  Don’t know …………………………… 8 | —**►982**  —**►982** |
| 980 | For how long did s/he have the change in the amount of urine passed daily? | Days ………………………….  Months ………………………..  Don’t know ……………............998 |  |
| 981 | Did s/he pass too much urine, too little urine, or no urine at all? | Too much …………………………….1  Too little ………………………………2  No urine at all ………………………..3  Don’t know …………………………...8 |  |
| 982 | During the illness that led to death, did s/he have any skin rash? | Yes ………………………………….….1  No …………………………………….. 2  Don’t know …………………………… 8 | —**►986**  —**►986** |
| 983 | For how long did s/he have the skin rash? | Days …………………………..  Don’t know …………………… 98 |  |
| 984 | Was the rash on:   1 The face? 2 The trunk? 3 The arms and legs? 4 Any other place? | **YES NO DK**  Face………………………1 2 8  Trunk……………………..1 2 8  Arms and Legs ………. 1 2 8  Other place ……………. 1 2 8  Specify _______________ |  |
| 985 | What did the rash look like? | Measles rash …………………………1  Rash with clear fluid …………………2  Rash with pus ………………………..3  Don’t know ……………………………8 |  |
| 986 | Did s/he have red eyes? | Yes ………………………………….….1  No …………………………………….. 2  Don’t know …………………………… 8 |  |
| 987 | Did s/he have bleeding from the nose, mouth, or anus? | Yes ………………………………….….1  No …………………………………….. 2  Don’t know …………………………… 8 |  |
| 988 | Did s/he ever have shingles/herpes zoster? | Yes ………………………………….….1  No …………………………………….. 2  Don’t know …………………………… 8 |  |
| 989 | Did s/he have weight loss? | Yes ………………………………….….1  No …………………………………….. 2  Don’t know …………………………… 8 | —**►990**  —**►990** |
| 989.1 | For how long did s/he have weight loss? | Days ………………………….  Months ………………………..  Don’t know ……………............98 |  |
| 989.2 | Did s/he look very thin and wasted? | Yes ………………………………….….1  No …………………………………….. 2  Don’t know …………………………… 8 |  |
| 990 | Did s/he have mouth sores or white patches in the mouth or on the tongue? | Yes ………………………………….….1  No …………………………………….. 2  Don’t know …………………………… 8 | —**►991**  —**►991** |
| 990.1 | For how long did s/he have mouth sores or white patches in the mouth or on the tongue? | Days …………………………..  Don’t know …………………… 9 8 |  |
| 991 | Did s/he have any swelling? | Yes ………………………………….….1  No …………………………………….. 2  Don’t know …………………………… 8 | —**►992**  —**►992** |
| 991.1 | For how long did s/he have the swelling? | Days ………………………….  Months ………………………..  Don’t know ……………............998 |  |
| 991.2 | Was the swelling on:   1 The face? 2 The joints? 3 The ankles? 4 The whole body? 5 Any other place? | **YES NO DK**  Face………………………1 2 8  Joints……………………..1 2 8  Ankles…………………. . 1 2 8  Whole body…………….. 1 2 8  Other place ……………. 1 2 8  Specify _______________ |  |
| 992 | Did s/he have any lumps? | Yes ………………………………….….1  No …………………………………….. 2  Don’t know …………………………… 8 | —**►993**  —**►993** |
| 992.1 | For how long did s/he have the lumps? | Days ………………………….  Months ………………………..  Don’t know ……………............998 |  |
| 992.2 | Were the lumps on:   1 The neck? 2 The armpit? 3 The groin? 4 Any other place? | **YES NO DK**  Neck………………………1 2 8  Armpit………………….. 1 2 8  Groin………………. …... 1 2 8  Other place ……………. 1 2 8  Specify _______________ |  |
| 993 | Did s/he have yellow discoloration of the eyes? | Yes ………………………………….….1  No …………………………………….. 2  Don’t know …………………………… 8 | —**►994**  —**►994** |

| 993.1 | For how long did s/he have yellow discoloration of the eyes? | Days ………………………….  Months ………………………..  Don’t know ……………............98 |  |
| --- | --- | --- | --- |
| 994 | Did s/he look pale (thinning/lack of blood) or have pale palms, eyes or nail beds? | Yes ………………………………….….1  No …………………………………….. 2  Don’t know …………………………… 8 | —**►995**  —**►995** |
| 994.1 | For how long did s/he look pale or have pale palms, eyes or nail beds? | Days ………………………….  Months ………………………..  Don’t know ……………............98 |  |
| 995 | Did s/he have an ulcer, abscess, or sore anywhere on the body? | Yes ………………………………….….1  No …………………………………….. 2  Don’t know …………………………… 8 | —**►1001**  —**►1001** |
| 995.1 | For how long did s/he have the ulcer, abscess, or sore? | Days …………………………..  Don’t know …………………… 9 8 |  |
| 995.2 | What was the location of the ulcer, abscess, or sore? | (Specify) |  |

| **SECTION 10. TREATMENT AND HEALTH SERVICE USE FOR THE FINAL ILLNESS** | | | | | |
| --- | --- | --- | --- | --- | --- |
| 1001 | | Did s/he receive any treatment for the illness that led to death? | | Yes ………………………………….….1  No …………………………………….. 2  Don’t know …………………………… 8 | —**►1100**  —**►1100** |
| 1002 | | Please tell me at which of the following places/facilities s/he received treatment during the illness that led to death:  1 Home?  2 Traditional healer?  3 Health post?  4 Government clinic?  5 Government health center?  6 Government hospital?  7 Private clinic?  8 private health center?  9 Private hospital?  10 Pharmacy, drug seller, store?  11 Any other placer or facility? | | **Yes No DK**  Home ………………… 1 2 8  Traditional healer…… 1 2 8  Health post………………1 2 8  Government clinic…... 1 2 8  Government health center..1 2 8  Government hospital…. 1 2 8  Private clinic ……………1 2 8  private health center……..1 2 8  Private hospital…………1 2 8  Pharmacy/drug seller  /store ……………….. 1 2 8  Other place __________1 2 8  Specify |  |
| 1002.1 | | Where did the person receive help for the first time | |  |  |
| 1002.2 | | Where did the person receive help for the second time | |  |  |
| 1002.3 | | Where did the person receive help for the third time | |  |  |
| 1003 | | Can you please list the drugs s/he was given for the illness that led to death?  COPY FROM PRESCRIPTION/DISCHARGE NOTES IF AVAILABLE | |  |  |
| 1004 | | What type of treatment did s/he receive:   1 Oral rehydration salts and/or intravenous fluids (drip) treatment? 2 Blood transfusion? 3 Treatment/food through a tube passed through the nose? 4 Any other treatment? | | **YES NO DK**  Ors/drip treatment………1 2 8  Blood transfusion……… 1 2 8  Through the nose……… 1 2 8  Other place ……………. 1 2 8  Specify _______________ |  |
| 1005 | In the month before death, how many contacts with formal health services did s/he have? | | Number of contacts………….  Don’t know …………………… 98 | |  |
| 1006 | Did a health care worker tell you the cause of death? | | Yes ………………………………….….1  No …………………………………….. 2  Don’t know …………………………… 8 | | —**►1008**  —**►1008** |
| 1007 | What did the health care worker say? | |  | |  |
| 1008 | Did s/he have any operation for the illness? | | Yes ………………………………….….1  No …………………………………….. 2  Don’t know …………………………… 8 | | —**►1101**  —**►1101** |
| 1009 | How long before death did s/he have the operation? | | Days …………………………..  Don’t know …………………… 98 | |  |
| 1010 | On what part of the body was the operation? | | Abdomen ………………………………1  Chest ………………………………......2  Head ……………………………………3  Other _________________________6  (Specify)  Don’t know …………………………….8 | |  |

| **SECTION 11. RISK FACTORS** | | | |
| --- | --- | --- | --- |
| 1101 | Did s/he drink alcohol? | Yes ………………………………….….1  No …………………………………….. 2  Don’t know …………………………… 8 | —**►1106**  —**►1106** |
| 1102 | How long had s/he been drinking?  RECORD 'OO IF LESS THAN ONE YEAR | year …………………………..  Don’t know …………………… 9 8 |  |
| 1103 | How often did s/he drink alcohol? | Daily ……………………………………1  Frequently (Weekly) ………………….2  Once in a while ……………………… 3  Don’t know ……………………………8 |  |
| 1104 | Did s/he stop drinking? | Yes ………………………………….….1  No …………………………………….. 2  Don’t know …………………………… 8 | —**►1106**  —**►1106** |
| 1105 | How long before death did s/he stop drinking?  RECORD 'OO IF LESS THAN ONE MONTH | Months ………………………..  Don’t know …………………… 9 8 |  |
| 1106 | Did s/he smoke tobacco (cigarette, cigar, pipe etc.)? | Yes ………………………………….….1  No …………………………………….. 2  Don’t know …………………………… 8 | —**►1112**  —**►1112** |
| 1107 | How long had s/he been smoking?  RECORD 'OO IF LESS THAN ONE YEAR | Years ………………………….  Don’t know …………………… 9 8 |  |
| 1108 | How often did s/he smoke? | Daily ……………………………………1  Frequently (Weekly) ………………….2  Once in a while ……………………… 3  Don’t know ……………………………8 |  |
| 1109 | How many cigarettes did s/he smoke daily? | Number of cigarettes……….  Don’t know …………………… 9 8 |  |
| 1110 | Did s/he stop smoking before death? | Yes ………………………………….….1  No …………………………………….. 2  Don’t know …………………………… 8 | —**►1112**  —**►1112** |
| 1111 | How long before death did s/he stop smoking? RECORD 'OO IF LESS THAN ONE MONTH | Months ………………………..  Don’t know …………………… 9 8 |  |
| 1112 | Did s/he chew chat? | Yes ………………………………….….1  No …………………………………….. 2  Don’t know …………………………… 8 | —**►1117**  —**►1117** |
| 1113 | How long had s/he been chewing chat?  RECORD 'OO IF LESS THAN ONE YEAR | year …………………………..  Don’t know …………………… 9 8 |  |
| 1114 | How often did s/he chew chat? | Daily ……………………………………1  Frequently (Weekly) ………………….2  Once in a while ……………………… 3  Don’t know ……………………………8 |  |
| 1115 | Did s/he stop chewing chat? | Yes ………………………………….….1  No …………………………………….. 2  Don’t know …………………………… 8 | —**►1117**  —**►1117** |
| 1116 | How long before death did s/he stop chewing? RECORD 'OO IF LESS THAN ONE MONTH | Months ………………………..  Don’t know …………………… 9 8 |  |
| 1117 | RECORD THE TIME AT THE END OF INTERVIEW | HOURS  MINUTES |  |

**INTERVIEWER’S OBSERVATIONS**

**TO BE FILLED IN AFTER COMPLETING INTERVIEW**

**______________________________________________________________________________________**______________________________________________________________________________________________________________________________________________________________________________________________________________________________________________________________________________________________________________________________________________________________________________________________________________________________________________

COMMENT ON SPECIFIC QUESTIONS:

**______________________________________________________________________________________**________________________________________________________________________________________________________________________________________________________________________________________________________________________________________________________________________________________________________________________________________________________

ANY OTHER COMMENTS:

______________________________________________________________________________________________________________________________________________________________________________________________________________________________________________________________________________________________________________________________________________________________________________________________________________________________________________

SUPERVISOR’S OBSERVATIONS

______________________________________________________________________________________________________________________________________________________________________________________________________________________________________________________________________________________________________________________________________________________________________________________________________________________________________________

__________________________________________________________________________________________________________________________________________________________________________________________________________________________________________________________________

NAME OF THE SUPERVISOR: ___________________________	DATE ______________________
