# Supplementary material for: Social determinants of adult mortality from non-communicable diseases in northern Ethiopia, 2009-2015: Evidence from health and demographic surveillance site
Source: PLoS One. 2017 Dec 13;12(12):e0188968. doi: 10.1371/journal.pone.0188968 (PMC5728486; doi:10.1371/journal.pone.0188968)
Supplement: S1 Data Collection tools — (ZIP) [file pone.0188968.s003.zip › S2 data collection tools/VA Questionnaire 3 Tigrigna.doc]

**ቪኤ ቃለ መሕተት 3:**

**ሞት ሰብ ዕድመ 15 ዓመትን ካብኡ ንላዕልን**

| **መግለፂ ስምምዕነት**  ጥዕና ይሃበለይ ከመይ ትሓድሩ/ትውዕሉ  ሽመይ ____________________________ ይበሃል ምስ መቐለ ዩኒቨርሲቲ እየ ዝሰርሕ ፣ እምባኣርከስ ሐዚ ኣብ ሕ/ሰብና ዝበፅሑ ዘለው ናይ ሞት ኩነታት ንምፍላጥ ሓበሬታታት እናኣከብና ኢና፡፡ ስለዝኾነ ናቶም/ናተን ምትሕብባርን ተሳታፋይነትን ብጣዕሚ ኢና እንደልዩ፡፡ ኣብ ገዝኦም ብዘሓለፈ ግዜ ብዛዕባ ዝሞተ/ተት ሰብ ንሞት ዘብቅዖ/ዓ ምኽንያት ሓደ ሓደ ሕቶታት ክንሓቶም/ተን ንደሊ ፡፡ ዝህቡና/ባና ዝኾነ ይኹን ሓበሬታ ብሚስጢር ከምንሕዞ ክነረጋግፀሎም ንፈቱ፡፡ ንፅንዓት ካብ ንደልዮ ወፃኢ ንዖኦም ወይ ንመዋቲ ንምፍልላይ ንጥቀመሉ ምንም ነገር ኣይህሉን፡፡ ኣብዚ ፅንዓት ዝሳተፉ ብናይ ባዕሎም ሰናይ ፈቓድ ስለዝኾነ ክምልስዎ/ኦ ዘይደልዩ/ያ ሕቶ እንተሃልዩ ናይ ዘይምምላስ መሰሎም/ለን ሕልው እዩ፡፡ ከምኡውን ኣብ ዝኾነ ሰዓት ነቲ ቃለ መሕተት ሙሉእ ብሙሉእ ናይ ምቁራፅ መሰል ኣለዎም/ወን፡፡ ስለዝኾነ ንዝህቡና/ባና ሓበሬታ መንግስቲ ንሕብረተሰብ ዝህቦም ኣገልግሎት ንምምሕያሽ ስለዝሕግዞ ንሶም/ሰን ኣብዚ ፅንዓት ብፍቓደኝነት ከምዝሳተፉ/ፋ ተስፋ ንገብር፡፡ ሐዚ ስለ እቲ ቓለ መሕተት ዓላማን ትሕዝቶን ዝሓቱኒ/ታኒ ሕቶ እንተሃልዩ እቲ ዕድል ንዖኦም/አን ክህቦም/በን፡፡ ሐዚ እቲ ቓለ መሕተት ክቕፅል'ዶ ይኽእል  ናይ ሓበሬታ ኣካቢ ክታም መዓልቲ  ተሓታቲ ንክሕተቱ/ታ ተሰማሚዖም እዮም…….. 1 ኣይተሰማምዑን……...2 ናይቲ ቃለመሕተት መወዳእታ  **🢃** | | | | | | |
| --- | --- | --- | --- | --- | --- | --- |
| **ክፍሊ 1 :** ውፅኢት **ርክብ ቪኤ** መረዳእታ ኣካባይ | | | | | | |
| ሽም ኣካቢ ሓበሬታ: ______________________ | | | ሽም ጣብያ __________________  ቁሸት _______________________ | | | መፍለዪ ቑፅሪ ስድራ(ቑፅሪ ገዛ):  ___________ |
| ክታም ኣካቢ ሓበሬታ : ______________________ | | |  |  |  |  |
| ሽም ተቖፃፃሪ ፡ ______________________ | | |  | | | |
| ክታም ተቖፃፃሪ ______________________ | | |  | | | |
| ውፅኢት 1^ይ^ ዑደት | 1.ተጠናቒቑ 2. ኣብ ገዝኦም/አን ኣይተረኸቡን/ባን 3. ተቛሪፁ 4. ፍቓደኛ ኣይኮኑን/ናን | | |  | ንኻልእ መዓልቲ ቖፀሮ ዝተትዘሉ ዕለት ______________________ | |
| ውፅኢት 2^ይ^ ዑደት | 1.ተጠናቒቑ 2. ኣብ ገዝኦም/አን ኣይተረኸቡን/ባን 3. ተቛሪፁ 4. ፍቓደኛ ኣይኮኑን/ናን | | |  | ንኻልእ መዓልቲ ቖፀሮ ዝተትዘሉ ዕለት ______________________ | |
| ውፅኢት 2^ይ^ ዑደት | 1.ተጠናቒቑ 2. ኣብ ገዝኦም/አን ኣይተረኸቡን/ባን 3. ተቛሪፁ 4. ፍቓደኛ ኣይኮኑን/ናን | | |  | ንኻልእ መዓልቲ ቖፀሮ ዝተትዘሉ ዕለት ______________________ | |
| ቃለ-መሕትት ዝተገብረሉ ዕለት ፡ ዕለት/ወርሒ/ዓ.ም | | ______/______/________ | | | | |

| **ክፍሊ 2 - ቀንዲ ሓበሬታታት ተሓታቲ** | | | |
| --- | --- | --- | --- |
| 201 | ቓለ መሕትት ዝተጀመረሉ እዋን መዝግብ/ቢ | ሰዓት ……………………….  ደቒቓ ………………….. |  |
| 202 | ሽም ተሓታቲ/ት | _____________________________  ሽም |  |
| 203 | ፆታ ተሓታቲ | ኣነስተይቲ …………………………….. 1  ተባዕታይ ……………………………… 2 |  |
| 204 | ዕድመ ተሓታቲ/ት | ዓመት |  |
| 205 | ምስ መዋቲ/ቲት ርክብኩም እንታይ እዩ? | ኣቦ/ኣዶ ………………… 1  ብዓልቲ ሓዳሩ/ብዓል ሓዳራ ……. …. 2  ሓው/ሓፍቲ……………..…………… 3  ካልእ ዘመድ /ይገለፅ/ ____________ 4  ዝምድና የብሎምን …………………… 5 |  |
| 206 | መዋቲ/ቲት ንሞት ክሳብ ዝበቕዐሉ ጊዜ ንስኹም/ኽን ንኽንደይ እዋን ሓቢርኩምዎ/ክንኦ ፀኒሕኹም/ኽን | መጀመርያ ሕማም ካብ ዝጅምሮ/ራ እዋን………………………………… 1  ድሕሪ ሕማም ምስጀመሮ/ራ………… 2  ቅድሚ ምሟቱ/ታ ቅድሚ ቁሩብ መዓልቲ …………………………… 3 |  |

| **ክፍሊ 3 - ሓበሬታ መዋትን፣ ዝሞታሉ/ትሉ ቦታን ዕለትን** | | | | | | | | | | | | | | | | | | | | | | | | | | |  | | | | | |
| --- | --- | --- | --- | --- | --- | --- | --- | --- | --- | --- | --- | --- | --- | --- | --- | --- | --- | --- | --- | --- | --- | --- | --- | --- | --- | --- | --- | --- | --- | --- | --- | --- |
| 301 | | ሽም መዋቲ/ቲት መን ይበሃል? | | | | | | | _____________________________  ሽም | | | | | | | | |  | | | | | | | | |  | | | | | |
| 302 | | ፆታ መዋቲ/ቲት | | | | | | | ኣነስተይቲ ……………………………… 1  ተባዕታይ …………………………………. 2 | | | | | | | | |  | | | | | | | | |  | | | | | |
| 303 | | መዋቲ/ት ዝተወለደሉ/ትሉ መዓዝ ነይሩ?  መዓልቲ ወይ ወርሒ እንድሕር ዘይፈልጥዎ 98 መዝግብ/ቢ፣ ዓመት እንተዘይፈሊጦምዎ 9998 መዝግብ/ቢ | | | | | | | ዕለት ………………………  ወርሒ ………………….  ዓመት …………………….. | | | | | | | | |  | | | | | | | | |  | | | | | |
| 304 | | እቲ/ታ መዋቲ/ት ዕድሚኡ/ኣ ክንደይ ነይሩ | | | | | | | ዕድመ ብዓመት?…………………… | | | | | | | | |  | | | | | | | | |  | | | | | |
| 305 | | እቲ/ታ መዋቲ/ቲት ዝሞተሉ/ትሉ እዋን መዓዝ ነይሩ? | | | | | | | ዕለት …………………………..  ወርሒ …………………………  ዓመት …………………….. | | | | | | | | |  | | | | | | | | |  | | | | | |
| 306 | | ኣበይ ቦታ ሞይቱ/ታ | | | | | | | ሆስፒታል …………………………………. 1  ጥዕና ጣብያ …………………………….… 2  ካልእ ጥዕና ትካል………....................……. 3  ገዛ……………………………………..……. 4  ካልእ /ይገለፅ/ ______________________ 5  ኣይፈልጦን…………………………………. 8 | | | | | | | | |  | | | | | | | | |  | | | | | |
| 307 | | ኣብ ሆስፒታል ወይ ኣብ ካልእ ትካል እንተሞይቱ/ታ ናይቲ ትካል ሽምን ኣድራሻን መዝግብ/ቢ | | | | | | | | | | | | | | | |  | | | | | | | | |  | | | | | |
| 308 | | ስርሑ/ሓ እንታይ ነይሩ፣ ብዋናነት ዝሰርሖ/ትሰርሖ ዝነበረ/ት ቀንዲ ስርሑ/ሓ እንታይ ነይሩ? | | | | | | | ሓረስታይ ………….................….… 1  ተምሃራይ/ሪት … …………………. 2  ነጋዳይ/ቲ ………….……………… 3  ሓላዊ/ት ከብቲ ……….………….. 4  መዓልታዊ ሰራሕተኛ/ናይ ገዛ ሰራሕተኛ 5  ናይ ገዛ ወሓለ …………….…….…… 6  መንግስቲ ሰራሕተኛ ………………… 7  ስራሕ ኣይነብሮን/ራን …………..……. 8  ካልእ (ይገለፅ)………………………… 9  ኣይፈልጦን ………………………..……… 98 | | | | | | | | |  | | | | | | | | |  | | | | | |
| 309 | | መዋቲ/ት ትምህርቲ ደረጅኡ/ኣ ክንደይ ወዲኡ/ኣ ነይሩ/ራ? | | | | | | | ዘይተምሃረ/ት …………..................... 1  ካብ 1^ይ^ – 4^ይ^ …………………....... 2  ካብ 5^ይ^ – 8^ይ^ …………………...... 3  ካብ 9^ይ^ – 10^ይ^ ………………........ 4  ካብ 11 – 12^ተ^ ………………...... 5  ኣይፈልጦን ………………………...... 8 | | | | | | | | |  | | | | | | | | |  | | | | | |
| 310 | | ኩነታት መርዓ መዋቲ/ት እንታይ ነይሩ? | | | | | | | ዘይተመርዓወ/ት ………………………….. 1  ተመርዒዩ/ያ ምስ ሓዳሩ/ራ ዝነብር/ትነብር … 2  ከይተመርዓው ሓቢሮም ዝነብሩ ……………. 3  ዝሞተቶ/ታ ……………………………….… 4  ዝተፋትሐ/ት ………………………………. 5  ዝተፈላለዩ ………………………………….. 6  ኣይፈልጦን ………………………………… 8 | | | | | | | | |  | | | | | | | | |  | | | | | |
|  | **ክፍሊ 4፡ ከም ኣገላልፃ ተሓታቲ መዋቲ/ት ንሞት ዘብቅዕዎ/ዋ ሕማማት ወይ ነገራት** | | | | | | | | | | | | | | | | | | | | | | | | | | |  | | | | |
|  | 401 | | በይዘኦም/አን እቲ/ታ መዋቲ/ት ንሞት ዘብቅዖ/ዓ ሕማም ወይ ኩነታት እንተዝነግሩኒ/ራኒ?  **መዘከርታ**፡ ብዝተኽአለ መጠን ኣፍቲ ከባቢ ዝጥቀምሉ መግለፂ ምልክታት ተጠቐም/ሚ  _______________________________________________________________________  ___________________________________________________________________  _______________________________________________________________________  ___________________________________________________________________  _______________________________________________________________________  ___________________________________________________________________  _______________________________________________________________________  ___________________________________________________________________  ______________­­­­­­_________________________________________________________  ___________________________________________________________________  _______________________________________________________________________  _________________________________________________________________ | | | | | | | | | | | | | | | | |  | | | | | | | |  | | | | |
|  | 402 | | ከም ኣገላልፃ ተሓታቲ/ት ቀዳማይ ደረጃ ምኽንያት ሞት  _________________________________________________________ | | | | | | | | | | | | | | | | |  | | | | | | | |  | | | | |
|  | 403 | | ከም ኣገላልፃ ተሓታቲ/ት ካልኣይ ደረጃ ምኽንያት ሞት  _________________________________________________________ | | | | | | | | | | | | | | | | |  | | | | | | | |  | | | | |
|  | **ክፍሊ 5 ፡ ታሪኽ ኣቐዲሙ ብሕክምና ዝፍለጥ ዝነበረ ፀገም ኩነታት ጥዕና መዋቲ/ት** | | | | | | | | | | | | | | | | | | | | | | | | | | |  | | | | |
|  | ሐዚ ድማ ናይዚ/ዛ መዋቲ/ት ውልቀሰብ ኣቀዲሞም ዝፍለጡ ዝነበሩ ናይ ጥዕና ፀገማት፣በፂሖምዎ/ዋ ዝነበሩ ሓደጋ/ጉድኣት፣ ከምኡዉን ሓሚሙ/ማ እናሃለወ/ት ዝነበርዎ/ዋ ምልክታት ብዝምልከት ሓደ ሓደ ሕቶታት ክሓቶም/ተን እየ፡፡ ሓደ ሓደ ሕቶታት ምስ ሞት መዋቲ/ት ብቐጥታ ርክብ ዘይክህልዎም ይኽእል'ዩ፡፡ በይዝኦም/አን ኩሎም ሕቶታት ንምምላስ ይተሓባበሩኒ/ራኒ፡፡ እዚ ድማ መዋቲ/ት ንዝነበርዎ/ዋ ሕማማት ትኽክለኛ መረዳእታ ክህበና ይኽእል እዩ፡፡  በይዝኦም/አን መዋቲ/ት ካብዞም ቀፂሎም ዘለዎ ሕማማት እንተነየሩዎ/ዋ ይሓብሩለይ/ራለይ፡፡ | | | | | | | | | | | | | | | | | | | | | | | | | | |  | | | | |
|  | 501 | | | | ደም በዝሒ /ምዉሳኽ ፀቕጢ ደም | | | | | | | እወ …………………………………… 1  ኣይፋሉን ……………………………… 2  ኣይፍለጥን …………………………… 8 | | | | | | |  | | | | | | | | |  | | | | |
|  | 502 | | | | ሕማም ሽኮርያ | | | | | | | እወ …………………………………… 1  ኣይፋሉን ……………………………… 2  ኣይፍለጥን …………………………… 8 | | | | | | |  | | | | | | | | |  | | | | |
|  | 503 | | | | ኣዝማ | | | | | | | እወ …………………………………… 1  ኣይፋሉን ……………………………… 2  ኣይፍለጥን …………………………… 8 | | | | | | |  | | | | | | | | |  | | | | |
|  | 504 | | | | ሕማም ባርያ/ዘውድቕ ሕማም | | | | | | | እወ …………………………………… 1  ኣይፋሉን ……………………………… 2  ኣይፍለጥን …………………………… 8 | | | | | | |  | | | | | | | | |  | | | | |
|  | 505 | | | | ሕፅረት ምግቢ | | | | | | | እወ …………………………………… 1  ኣይፋሉን ……………………………… 2  ኣይፍለጥን …………………………… 8 | | | | | | |  | | | | | | | | |  | | | | |
|  | 506 | | | | ካንሰር/ነቀርሳ | | | | | | | እወ …………………………………… 1  ኣይፋሉን ……………………………… 2  ኣይፍለጥን …………………………… 8 | | | | | | | —**►508**  —**►508** | | | | | | | | |  | | | | |
|  | 507 | | | | እቲ ካንሰር ኣበይናይ ናይ ሰውነቱ/ታ ክፍሊ ከምዝነበረ ይገለፅ? | | | | | | | ዓይነት/ክፍሊ  _________________________________ | | | | | | |  | | | | | | | | |  | | | | |
|  | 508 | | | | ሕማም ዓባይ ሰዓል | | | | | | | እወ …………………………………… 1  ኣይፋሉን ……………………………… 2  ኣይፍለጥን …………………………… 8 | | | | | | |  | | | | | | | | |  | | | | |
|  | 509 | | | | ኤች.ኣይ.ቪ/ኤድስ? | | | | | | | እወ …………………………………… 1  ኣይፋሉን ……………………………… 2  ኣይፍለጥን …………………………… 8 | | | | | | |  | | | | | | | | |  | | | | |
|  | 510 | | | | ብሕክምና ዝተረጋገፀ ካልእ ሕማም ነይርዎ/ዋ'ዶ? | | | | | | | እወ …………………………………… 1  ኣይፋሉን ……………………………… 2  ኣይፍለጥን …………………………… 8 | | | | | | | —**►601**  —**►601** | | | | | | | | |  | | | | |
|  | 511 | | | | እቲ ሕማም ክነግሩኒ/ራኒ ይኽእሉ/ላ'ዶ? | | | | | | | ዓይነት እቲ ሕማም | | | | | | |  | | | | | | | | |  | | | | |
|  | **ክፍሊ 6 ፡ ታሪኽ ጉድኣት/ሓደጋታት** | | | | | | | | | | | | | | | | | | | | | | | | | | |  | | | | |
|  | 601 | | | እቲ/ታ መዋቲ/ት ንሞት ዘብቅዕ ጉድኣት/ሓደጋ በፂሕዎ/ዋ'ዶ ነይሩ? | | | | | | | | | እወ …………………………………… 1  ኣይፋሉን ……………………………… 2  ኣይፍለጥን …………………………… 8 | | | | | | | | —**►604**  —**►604** | | | | | | |  | | | | |
|  | 602 | | | እቲ/ታ መዋቲ/ት እንታይ ዓይነት ጉድኣት/ሓደጋ በፂሕዎ/ዋ ነይሩ? | | | | | | | | | ሓደጋ ተሽከርከርቲ ………… 01 ምውዳቕ…………………………… 02 ምጥላቕ …………………………. 03 ምምራዝ…………………….. … 04 ምቅፃል …………………………… 05 ምድብዳብ/ጥቕዓት……………….... 06 ካልእ /ይነፀር/ _________________ 96  ኣይፈልጥን ……………………… 98 | | | | | | | |  | | | | | | |  | | | | |
|  | 603 | | | እቲ ጉድኣት/ሓደጋ ዝበፀሖ/ሓ ብኻልኦት ሰባት ኮነ ተባሂሉ ዝተፈፀመ ድዩ ነይሩ? | | | | | | | | | እወ …………………………………… 1  ኣይፋሉን ……………………………… 2  ኣይፍለጥን …………………………… 8 | | | | | | | | —**►607** | | | | | | |  | | | | |
|  | 604 | | | መዋቲ/ት ዓርሰ ቅትለት ዝፈፀመ/ት ይመስሎም/ለን'ዶ ? | | | | | | | | | እወ …………………………………… 1  ኣይፋሉን ……………………………… 2  ኣይፍለጥን …………………………… 8 | | | | | | | |  | | | | | | |  | | | | |
|  | 605 | | | እቲ/ታ መዋቲ/ት ንሞት ዘብቅዖ/ዓ ብእንስሳ ተነኺሱ/ሳ ወይ ብነፍሳት ተነኺሱ/ሳ'ዶ ይመስሎም/ለን? | | | | | | | | | እወ …………………………………… 1  ኣይፋሉን ……………………………… 2  ኣይፍለጥን …………………………… 8 | | | | | | | | —**►607**  —**►607** | | | | | | |  | | | | |
|  | 606 | | | እንታይ ዓይነት እንስሳ/ነፍሳት? | | | | | | | | | ከልቢ…………………………………… 1  ተበን …………………………………. 2  ነፍሳት …………………………………. 3  ካልእ /ይነፀር/ _________________ 6  ኣይፍለጥን …………………………… 8 | | | | | | | |  | | | | | | |  | | | | |
|  | 607 | | | ሕቶ ተራ ቑፅሪ 302 ርኢኻ/ኪ ናይ መዋቲ ፆታ አረጋግፅ/ፂ:    ኣነስተይቲ ተባዕታይ  701 | | | | | | | | | | | | | | | | | 901 | | | | | | |  | | | | |
|  | **ክፍሊ 7 ፡ ምስ ናይ ደቅኣንስትዮ ሕማማት ዝተተሓሓዙ ሓበሬታትን ምልክታትን** | | | | | | | | | | | | | | | | | | | | | | | | | | |  | | | | |
|  | 701 | | | | | ሕበጥ ጡብ ወይ ቁስለት ነይርዋ'ድዩ? | | | | | | | | እወ …………………………………… 1  ኣይፋሉን ……………………………… 2  ኣይፍለጥን …………………………… 8 | | | | | | | | —**►703**  —**►703** | | | | | |  | | | | |
|  | 702 | | | | | እቲ ቁስለት ወይ ሕብጠት ጡብ ንኽንደይ እዋን ዝኣክል ምስኣ ፀኒሑ? | | | | | | | | መዓልቲ ……………………….  ወርሒ ………………………..  ኣይፈልጦን …………….......... 98 | | | | | | | |  | | | | | |  | | | | |
|  | 703 | | | | | ኣብ እዋን ወርሓዊ ፅግያት ብርቱዕ ደም ብማህፀን ይፈሳ'ዶ ነይሩ? | | | | | | | | እወ …………………………………… 1  ኣይፋሉን ……………………………… 2  ኣይፍለጥን …………………………… 8 | | | | | | | | —**►705**  —**►705** | | | | | |  | | | | |
|  | 704 | | | | | ንኽንደይ እዋን ዝኣክል ኣብ እዋን ወርሓዊ ፅግያታ ብርቱዕ ደም ብማህፀና ይፈሳ ነይሩ? | | | | | | | | መዓልቲ ……………………….  ወርሒ ………………………..  ኣይፈልጦን …………….......... 98 | | | | | | | |  | | | | | |  | | | | |
|  | 705 | | | | | ኣብ መንጎ ወርሓዊ ፅግያት ብማህፀና ደም ይፈሳ ነይሩ ድዩ? | | | | | | | | እወ …………………………………… 1  ኣይፋሉን ……………………………… 2  ኣይፍለጥን …………………………… 8 | | | | | | | | —**►707**  —**►707** | | | | | |  | | | | |
|  | 706 | | | | | ኣብ መንጎ ወርሓዊ ፅግያት ንኽንደይ ጊዜ ዝኣክል ደም ብማህፀና ይፈሳ ነይሩ? | | | | | | | | መዓልቲ ……………………….  ወርሒ ………………………..  ኣይፈልጦን ……………..........98 | | | | | | | |  | | | | | |  | | | | |
|  | 707 | | | | | መዋቲት ዘይተለመደ ብማህፀን ዝወፅእ ፈሳሲ ነይሩዋ ድዩ? | | | | | | | | እወ …………………………………… 1  ኣይፋሉን ……………………………… 2  ኣይፍለጥን …………………………… 8 | | | | | | | | **►801**  **►801** | | | | | |  | | | | |
|  | 708 | | | | | እዚ ዘይተለመደ ብማህፀን ዝወፅእ ፈሳሲ ንኽንደይ ጊዜ ፀኒሑ ነይሩ? | | | | | | | | መዓልቲ ……………………….  ወርሒ ………………………..  ኣይፈልጦን ……………..........98 | | | | | | | |  | | | | | |  | | | | |
|  | **ክፍሊ 8 ፡ ምስ ጥንሲ ዝተተሓሓዙ ሓበሬታን ምልክታትን** | | | | | | | | | | | | | | | | | | | | | | | | | | | | | | | |
|  | 801 | | | | | | መዋቲት ክትሞት እንተላ ጥንስቲ ነይራ ድያ? | | | | እወ ………………………1  ኣይፋሉን …………………2  ኣይፍለጥን ……………….8 | | | | | | —**►806**  —**►806** | | | | | | | | | | | | | | | |
|  | 802 | | | | | | ጥንሳ ክንደይ ገይሩ ነይሩ? | | | | ሰሙን …………………….  ወርሒ ……………………  ኣይፈልጦን ……………..........98 | | | | | |  | | | | | | | | | | | | | | | |
|  | 803 | | | | | | መዋቲት እዚ ጥንሲ እዙይ ሓዊስካ ክንደይ ጊዜ ጠኒሳ ነይራ? | | | | ቑፅሪ………………………….  ኣይፈልጦን …………………… 98 | | | | | |  | | | | | | | | | | | | | | | |
|  | 804 | | | | | | ኣብ ዝሓለፉ 3 ናይ ጥንሲ ኣዋርሕ ካብዞም ዝስዕቡ ሕማማት ኣይኒኦም ኣጋጢመምዋ ነይሮም፡   1 ብማህፀን ደም ምፍሳስ? 2 ሕማቕ ጨና ዘለዎ ብማህፀን ዝወፅእ ፈሳሲ? 3 ሕበጥ ገፅ ? 4 ሕማም ርእሲ? 5 ምዛር ርእሲ ? 6 ምንቅጥቃጥ? 7 ረስኒ ዘለዎ ሕማም ? 8 ካብ ወሊድ ወፃኢ ዝኾነ ሕሱም ሕማም ከብዲ ? 9 ገፃ ተቐይሩ/ሓሙኽሽቲ መሲላ ምስ ምሕፃር እስትንፋስ /ክልቲኡ ብሓባር/ ? 10 ካልእ ሕማም ነይሩዋ'ዶ? | | | **እወ ኣይፋል. ኣይፈልጥ ንእወ መዓዝ ወርሒ**  መድመይቲ ማህፀን…1 …..2………… 8 ___    ሕማቕ ጨና ዘለዎ ካብ ማህፀን  ዝወፅእ ፈሳሲ………..1…….2………….8 ___  ሕበጥ ገፅ ..………1……..2…………8 ___  ሕማም ርእሲ ……..1……..2……….. 8 ___  ምዛር ርእሲ ……….1……..2………...8 ___  ምንቅጥቃጥ ………...1……..2………...8 ___  ረስኒ ዘለዎ ሕማም …1……..2……….. 8 ___  ሕሱም ቁርፀት ከብዲ (ካብ ሕማም ሕርሲ ወፃኢ)1….2………..8 ___  ምምንጫሕ ምስ  ሕፅረት እስትንፋስ(ብሓባር)1….2………..8 ___  ካልእ ሕማም ……………1…2………..8 ___   (ይገለፅ) | | | | | | | | | | | | | | | |  | | | | | | |
|  | 805 | | | | | | ሕርሲ ተታሒዛ እናሃለወት ከይወለደት ድያ ሞይታ? | | | እወ …………………………………… 1  ኣይፋሉን ……………………………… 2  ኣይፍለጥን …………………………… 8 | | | | | | | | | | | | | | | |  | | | | | | |
|  | 806 | | | | | | መዋቲት ኣብ ቀረባ ጊዜ ወሊዳ'ዶ ነይራ? | | | እወ …………………………………… 1  ኣይፋሉን ……………………………… 2  ኣይፍለጥን …………………………… 8 | | | | | | | | | | | | | | | | **►816**  **►816** | | | | | | |
|  | 807 | | | | | | ምስ ወለደት ድሕሪ ክንደይ መዓልቲ ሞይታ? | | | መዓልቲ ……………………  ወርሒ ………………………..  ኣይፈልጦን ……………..........98 | | | | | | | | | | | | | | | |  | | | | | | |
|  | 808 | | | | | | ቆልዓ ቅድሚ ምውላዳ ኣብ እዋን ሕርሲ ካብ መጠን ንላዕሊ ደም ፈሲስዋ'ዶ ነይሩ? | | | እወ …………………………………… 1  ኣይፋሉን ……………………………… 2  ኣይፍለጥን …………………………… 8 | | | | | | | | | | | | | | | |  | | | | | | |
|  | 809 | | | | | | ቆልዓ ምስወለደት ካብ መጠን ንላዕሊ ደም ፈሲስዋ'ዶ ነይሩ? | | | እወ …………………………………… 1  ኣይፋሉን ……………………………… 2  ኣይፍለጥን …………………………… 8 | | | | | | | | | | | | | | | |  | | | | | | |
|  | 810 | | | | | | መዳሕንቲ ኣይወፅእን ኢሉ አሸጊርዋ'ዶ ነይሩ? | | | እወ …………………………………… 1  ኣይፋሉን ……………………………… 2  ኣይፍለጥን …………………………… 8 | | | | | | | | | | | | | | | |  | | | | | | |
|  | 811 | | | | | | ኣብ ሕርሲ ዶንጊያ'ዶ ነይራ (ልዕሊ 12 ሰዓት)? | | | እወ …………………………………… 1  ኣይፋሉን ……………………………… 2  ኣይፍለጥን …………………………… 8 | | | | | | | | | | | | | | | |  | | | | | | |
|  | 812 | | | | | | በየናይ ዓይነት መንገዲ ኣወላልዳ እያ ወሊዳ ነይራ ? | | | ብማህፀን (ልሙድ ኣወላልዳ) …………1  ብመጥባሕቲ …………………………….2  ብመሳርሒ ተጎቲቱ………………………3  ካልእ ______________________......6  (ይገለፅ)  ኣይፈልጦን ……………………………. 8 | | | | | | | | | | | | | | | |  | | | | | | |
|  | 813 | | | | | | ሕማቕ ጨና ዘለዎ ናይ ማህፀን ፈሳሲ ነይሩዋ ድዩ? | | | እወ …………………………………… 1  ኣይፋሉን ……………………………… 2  ኣይፍለጥን …………………………… 8 | | | | | | | | | | | | | | | |  | | | | | |  |
|  | 814 | | | | | | ኣበይ እያ ወሊዳ ነይራ? | | | ሆስፒታል …………………………… 1  ጥዕና ጣብያ…………………………. 2  ጥዕና ኬላ…………………………….. 3  ክሊኒክ……………..……....................... 4  ገዛ…………………………………... 5  ካልእ /ይነፀር/ _________________ 6  ኣይፍለጥን…………………………… 8 | | | | | | | | | | | | | | | |  | | | | | |  |
|  | 815 | | | | | | መንዩ ኣዋሊድዋ ነይሩ? | | | ሓኪም /ዶክተር/ ……………………… 1  ነርስ/መዋልዳን/………………………… 2  ልምዲ መዋልዳን …………… 3  ዘመድ ……………………………….. 4  ባዕላ ……………………. 5  ካልእ /ይነፀር/ _________________ 6  ኣይፍለጥን…………………………… 8 | | | | | | | | | | | | | | | |  | | | | | |  |
|  | 816 | | | | | | ቅድሚ ምሟታ ቅድሚ ቁሩብ እዋን ጥንሲ ከይድዋ'ዶ ነይሩ? | | | እወ …………………………………… 1  ኣይፋሉን ……………………………… 2  ኣይፍለጥን …………………………… 8 | | | | | | | | | | | | | | | | **►901**  **►901** | | | | | |  |
|  | 817 | | | | | | ቅድሚ ምሟታ ቅድሚ ክንደይ መዓልቲ ነይሩ ጥንሲ ዝኸዳ? | | | መዓልቲ ………………………….  ኣይፍለጥን …………………… 98 | | | | | | | | | | | | | | | |  | | | | | |  |
|  | 818 | | | | | | እቲ ዝኸዳ ጥንሲ ክንደይ ወርሒ ገይሩ ነይሩ? | | | ወርሒ ……………………….  ኣይፍለጥን …………………… 98 | | | | | | | | | | | | | | | |  | | | | | |  |
|  | 819 | | | | | | ሂወታ ዝሓለፈ ጥንሲ ክተውርድ እንተላ ድዩ? | | | እወ …………………………………… 1  ኣይፋሉን ……………………………… 2  ኣይፍለጥን …………………………… 8 | | | | | | | | | | | | | | | |  | | | | | |  |
|  | 820 | | | | | | ጥንሲ ምሰውረደት ከቢድ መድመይቲ ነይሩዋ ድዩ? | | | እወ …………………………………… 1  ኣይፋሉን ……………………………… 2  ኣይፍለጥን …………………………… 8 | | | | | | | | | | | | | | | |  | | | | | |  |
|  | 821 | | | | | | እቲ ጥንሲ ከይተነኻኽአ ባዕሉ ድዩ ከይዱ? | | | እወ …………………………………… 1  ኣይፋሉን ……………………………… 2  ኣይፍለጥን …………………………… 8 | | | | | | | | | | | | | | | | **►901**  **►901** | | | | | |  |
|  | 822 | | | | | | እቲ ጥንሲ ንምውራድ መድሓኒት ወይ ካሊእ ሕክምና ተጠቒማ'ዶ ነይራ? | | | እወ …………………………………… 1  ኣይፋሉን ……………………………… 2  ኣይፍለጥን …………………………… 8 | | | | | | | | | | | | | | | |  | | | | | |  |
|  | **ክፍሊ 9 ፡ መዋቲ/ት ኣብ መወዳእታ እዋን ሕማም ዘርኣዮ/የቶ ምልክታት** | | | | | | | | | | | | | | | | | | | | | | | | | | | | | |  | |
|  | 901 | | | | | | መዋቲ/ት ቅድሚ ምሟቱ/ታ ንኽንደይ ጊዜ ሓሚሙ/ማ ነይሩ/ራ? | | | | | | | | መዓልቲ ……………………  ወርሒ ………………………..  ኣይፈልጦን ……………..........98 | | | | | | | | |  | | | | | | |  | |
|  | 902 | | | | | | መዋቲ/ት ረስኒ ነይርዎ/ዋ ድዩ? | | | | | | | | እወ …………………………………… 1  ኣይፋሉን ……………………………… 2  ኣይፍለጥን …………………………… 8 | | | | | | | | | **►906**  **►906** | | | | | | |  | |
|  | 903 | | | | | | እቲ ረስኒ ንኽንደይ ጊዜ ፀኒሑ ነይሩ? | | | | | | | | መዓልቲ ………………………  ወርሒ ………………………..  ኣይፈልጦን ……………..........98 | | | | | | | | |  | | | | | | |  | |
|  | 904 | | | | | | እቲ ረስኒ ኩሉ ጊዜ ድዩ ነይሩ ወይስ ሓልሓሊፉ? | | | | | | | | ኩሉ ጊዜ ………………………… 1  ሓልሓሊፉ …………………………. 2  ኣይፍለጥን ……………………… 8 | | | | | | | | |  | | | | | | |  | |
|  | 905 | | | | | | እቲ ረስኒ ምሸት ምሸት ጥራሕ ድዩ ነይሩ? | | | | | | | | እወ …………………………………… 1  ኣይፋሉን ……………………………… 2  ኣይፍለጥን …………………………… 8 | | | | | | | | |  | | | | | | |  | |
|  | 906 | | | | | | መዋቲ/ት ቑሪ ቑሪ እናበለ የንቀጥቅጦ/ጣ'ዶ ነይሩ እዩ? | | | | | | | | እወ …………………………………… 1  ኣይፋሉን ……………………………… 2  ኣይፍለጥን …………………………… 8 | | | | | | | | |  | | | | | | |  | |
|  | 907 | | | | | | መዋቲ/ት ሰዓል ነይሩዎ/ዋ ድዩ? | | | | | | | | እወ …………………………………… 1  ኣይፋሉን ……………………………… 2  ኣይፍለጥን …………………………… 8 | | | | | | | | | **►913**  **►913** | | | | | | |  | |
|  | 908 | | | | | | እቲ ሰዓል ንኽንደይ እዋን ፀኒሑ ነይሩ? | | | | | | | | መዓልቲ ……………………  ወርሒ ………………………..  ኣይፈልጦን ……………..........98 | | | | | | | | |  | | | | | | |  | |
|  | 909 | | | | | | እቲ ሰዓል ሓያል'ዶ ነይሩ? | | | | | | | | እወ …………………………………… 1  ኣይፋሉን ……………………………… 2  ኣይፍለጥን …………………………… 8 | | | | | | | | |  | | | | | | |  | |
|  | 910 | | | | | | እቲ ሰዓል ዓኽታ ነይሩዎ ድዩ? | | | | | | | | እወ …………………………………… 1  ኣይፋሉን ……………………………… 2  ኣይፍለጥን …………………………… 8 | | | | | | | | |  | | | | | | |  | |
|  | 911 | | | | | | እቲ ዓኽታ ደም ይሓውስ 'ዶ ነይሩ'ዩ? | | | | | | | | እወ …………………………………… 1  ኣይፋሉን ……………………………… 2  ኣይፍለጥን …………………………… 8 | | | | | | | | |  | | | | | | |  | |
|  | 912 | | | | | | ለይቲ ለይቲ የርሕፆ/ፃ'ዶ ነይሩ? | | | | | | | | እወ …………………………………… 1  ኣይፋሉን ……………………………… 2  ኣይፍለጥን …………………………… 8 | | | | | | | | |  | | | | | | |  | |
|  | 913 | | | | | | ሕፅረት ምትንፋስ 'ዶ የጋጥሞ/ማ ነይሩ'ዩ? | | | | | | | | እወ …………………………………… 1  ኣይፋሉን ……………………………… 2  ኣይፍለጥን …………………………… 8 | | | | | | | | | **►918**  **►918** | | | | | | |  | |
|  | 914 | | | | | | እቲ ሕፅረት ምትንፋስ ንኽንደይ ጊዜ ፀኒሑ ነይሩ? | | | | | | | | መዓልቲ ……………………  ወርሒ ………………………..  ኣይፈልጦን ……………..........98 | | | | | | | | |  | | | | | | |  | |
|  | 915 | | | | | | ብሕፅረት ምትንፋስ ዝተልዓለ መዓልታዊ ናይ ስራሕ ተግባሩ/ራ ንምስራሕ የፀግመሉ/ላ'ዶ ነይሩ? | | | | | | | | እወ …………………………………… 1  ኣይፋሉን ……………………………… 2  ኣይፍለጥን …………………………… 8 | | | | | | | | |  | | | | | | |  | |
|  | 916 | | | | | | ጥንቅልዒት (ብዝባኑ/ና) ደቂሱ/ሳ ሕፅረት ምትንፋስ የጋጥሞ/ማ ዶ ነይሩ? | | | | | | | | እወ …………………………………… 1  ኣይፋሉን ……………………………… 2  ኣይፍለጥን …………………………… 8 | | | | | | | | |  | | | | | | |  | |
|  | 917 | | | | | | መዋቲ/ት ክትንፍስ እንከሎ/ላ ሕፅፅ ሕፅፅ ዝብል ድምፂ ነይሩዎ/ዋ ድዩ? | | | | | | | | እወ …………………………………… 1  ኣይፋሉን ……………………………… 2  ኣይፍለጥን …………………………… 8 | | | | | | | | |  | | | | | | |  | |
|  | 918 | | | | | | መዋቲ/ት ናይ ደረት ሕማም( ውግኣት) ነይሩዎ/ዋ ድዩ? | | | | | | | | እወ …………………………………… 1  ኣይፋሉን ……………………………… 2  ኣይፍለጥን …………………………… 8 | | | | | | | | | **►928**  **►928** | | | | | | |  | |
|  | 919 | | | | | | ንኽንደይ ጊዜ ዝፀነሐ ናይ ደረት ሕማም(ውግኣት) ነይሩ'ዎ/ዋ? | | | | | | | | መዓልቲ ……………………  ወርሒ ………………………..  ኣይፈልጦን ……………..........98 | | | | | | | | |  | | | | | | |  | |
|  | 920 | | | | | | እቲ ናይ ደረት ሕማም(ውግኣት) ብሃንደበት ድዩስ ወይስ ቀስ ብቐስ ዝመፀ እዩ ነይሩ? | | | | | | | | ብሃንደበት……………………….…. 1  ቀስ ብቐስ……………………………… 2  ኣይፍለጥን ………………………… 8 | | | | | | | | |  | | | | | | |  | |
|  | 921 | | | | | | ሓያል ናይ ደረት ሕማም (ውግኣት) እንትለዓሎ/ላ እንተሎ እቲ ሕማም ክንደይ ጊዜ ይፀንሕ ነይሩ? | | | | | | | | ትሕቲ ፍርቂ ሰዓት………………. 1  ካብ ፍርቂ ሰዓት ክሳብ 24 ሰዓታት…. .2  ልዕሊ 24 ሰዓታት ………………... 3  ኣይፍለጥን ………………………… 8 | | | | | | | | |  | | | | | | |  | |
|  | 922 | | | | | | እቲ ናይ ደረት ሕማም (ውግኣት) ኣብ ትሕቲ ዓፅሚ ኣፍ ልቢ ድዩ ነይሩ? | | | | | | | | እወ …………………………………… 1  ኣይፋሉን ……………………………… 2  ኣይፍለጥን …………………………… 8 | | | | | | | | |  | | | | | | |  | |
|  | 923 | | | | | | እቲ ናይ ደረት ሕማሙ/ማ (ውግኣት) ኣብ ከባቢ ኣፍ ልቢ ኮይኑ ናብ ፀጋማይ ኩርንዑ/ዓ ዝኸድ ዓይነት 'ዶ ነየሩ? | | | | | | | | እወ …………………………………… 1  ኣይፋሉን ……………………………… 2  ኣይፍለጥን …………………………… 8 | | | | | | | | |  | | | | | | |  | |
|  | 924 | | | | | | እቲ ሕማሙ/ማ(ውግኣት) ኣብ ጎድኒ ዓፅሚ ከባቢ ድዩ ነይሩ? | | | | | | | | እወ …………………………………… 1  ኣይፋሉን ……………………………… 2  ኣይፍለጥን …………………………… 8 | | | | | | | | |  | | | | | | |  | |
|  | 925 | | | | | | እቲ ናይ ደረት ሕማም (ውግኣት) ዘየቋርፅ ድዩ ወይስ ሓልሓሊፉ ዩ ነይሩ? | | | | | | | | ዘየቋርፅ(ኩሉ ጊዜ) ………………………1  ሓልሓሊፉ …………………………. 2  ኣይፍለጥን ……………………… 8 | | | | | | | | |  | | | | | | |  | |
|  | 926 | | | | | | መዋቲ/ት እቲ ናይ ደረት ሕማሙ/ማ (ውግኣት) ክስዕል/ክትስዕል እንተሎ/እንተላ ይብእሶ/ሳ'ዶ ነይሩ? | | | | | | | | እወ …………………………………… 1  ኣይፋሉን ……………………………… 2  ኣይፍለጥን …………………………… 8 | | | | | | | | |  | | | | | | |  | |
|  | 927 | | | | | | መዋቲ/ት ትርግታ ልቡ/ባ ይስመዖ/ዓ'ዶ ነይሩ? | | | | | | | | እወ …………………………………… 1  ኣይፋሉን ……………………………… 2  ኣይፍለጥን …………………………… 8 | | | | | | | | |  | | | | | | |  | |
|  | 928 | | | | | | ውፅኣት ነይሩዎ/ዋ'ዶ? | | | | | | | | እወ …………………………………… 1  ኣይፋሉን ……………………………… 2  ኣይፍለጥን …………………………… 8 | | | | | | | | | **►933**  **►933** | | | | | | |  | |
|  | 929 | | | | | | እቲ ውፅኣት ንኽንደይ ጊዜ ፀኒሑ ነይሩ? | | | | | | | | መዓልቲ ……………………  ወርሒ ………………………..  ኣይፈልጦን ……………..........98 | | | | | | | | |  | | | | | | |  | |
|  | 930 | | | | | | እቲ ውፅኣት ሓልሓሊፉ ድዩ ወይስ ኩሉ ጊዜ ነይሩ? | | | | | | | | ኩሉ ጊዜ …………………………… 1  ሓልሓሊፉ …………………………. 2  ኣይፍለጥን ……………………… 8 | | | | | | | | |  | | | | | | |  | |
|  | 931 | | | | | | ሓሚሙ/ማ ኣብ ዝነበረሉ/ትሉ ጊዜ እቲ ፍታኑ/ና ደም ነይርዎ ድዩ? | | | | | | | | እወ …………………………………… 1  ኣይፋሉን ……………………………… 2  ኣይፍለጥን …………………………… 8 | | | | | | | | |  | | | | | | |  | |
|  | 932 | | | | | | እቲ ተቕማጥ ክብእሶ/ሳ እንተሎ ኣብ መዓልቲ ክንደይ ጊዜ የውፅኦ/ኣ ነይሩ? | | | | | | | | ብቑፅሪ……………………….  ኣይፍለጥን ……………………… 98 | | | | | | | | |  | | | | | | |  | |
|  | 933 | | | | | | መዋቲ/ት ተምላስ ነይሩዎ/ዋ'ዶ? | | | | | | | | እወ …………………………………… 1  ኣይፋሉን ……………………………… 2  ኣይፍለጥን …………………………… 8 | | | | | | | | | **►937**  **►937** | | | | | | |  | |
|  | 934 | | | | | | እቲ ተምላስ ንኽንደይ ጊዜ ፀኒሑ ነይሩ? | | | | | | | | መዓልቲ ……………………  ወርሒ ………………………..  ኣይፈልጦን ……………..........98 | | | | | | | | |  | | | | | | |  | |
|  | 935 | | | | | | እቲ ተምላስ ቡና ወይ ፅሩይ ደም ዝመስል ድዩ ነይሩ ወይስ ካልእ ዓይነት እዩ ነይሩ? | | | | | | | | ቡና ዝመስል ሕብሪ ዘለዎ ፈሳሲ ….. 1  ፅሩይ ደም ………………...……….. 2  ካልእ ዓይነት ___________________ 6  (ይገለፅ)  ኣይፍለጥን ………………… 8 | | | | | | | | |  | | | | | | |  | |
|  | 936 | | | | | | እቲ ተምላስ ክገዶ/ዳ እንተሎ ኣብ መዓልቲ ክንደይ ሻዕ የምልሶ/ሳ ነይሩ? | | | | | | | | ብቑፅሪ……………………….  ኣይፍለጥን ………………………98 | | | | | | | | |  | | | | | | |  | |
|  | 937 | | | | | | ሕቶ ተራ ቑፅሪ 302 ርኢኻ/ኺ ናይ መዋቲ ፆታ:  ኣንስታይ ተባዕታይ | | | | | | | | | | | | | | | | | **939** | | | | | | |  | |
|  | 938 | | | | | | ሕቶ ተራ ቑፅሪ 801,805,819 ርኢኻ/ኺ መዋቲት ዝሞተት ኣብ ሕርሲ፣ ወሊድ፣ ጥንሲ ክተውርድ እንተላ ወይ ድማ ድሕሪ ወሊድ  ኣይፋሉን እወ | | | | | | | | | | | | | | | | | **948** | | | | | | |  | |
|  | 939 | | | | | | መዋቲ/ት ቁርፀት ከብዲ ነይሩዎ/ዋ'ዶ? | | | | | | | | እወ …………………………………… 1  ኣይፋሉን ……………………………… 2  ኣይፍለጥን …………………………… 8 | | | | | | | | | **►941**  **►941** | | | | | | |  | |
|  | 940 | | | | | | ንኽንደይ ጊዜ ዝኣክል እቲ ቁርፀት ከብዲ ፀኒሑ ነይሩ? | | | | | | | | መዓልቲ ……………………  ወርሒ ………………………..  ኣይፈልጦን ……………..........98 | | | | | | | | |  | | | | | | |  | |
|  | 941 | | | | | | መዋቲ/ት ከብዱ/ዳ ይንፋሕ'ዶ ነይሩ? | | | | | | | | እወ …………………………………… 1  ኣይፋሉን ……………………………… 2  ኣይፍለጥን …………………………… 8 | | | | | | | | | **►945**  **►945** | | | | | | |  | |
|  | 942 | | | | | | እቲ መንፋሕቲ ከብዲ ንኽንደይ ጊዜ ዝኣክል ፀኒሑ ነይሩ? | | | | | | | | መዓልቲ ……………………  ወርሒ ………………………..  ኣይፈልጦን …………….......... 98 | | | | | | | | |  | | | | | | |  | |
|  | 943 | | | | | | ኣጀማምራ እቲ መንፋሕቲ ከብዲ ከመይ ነይሩ? | | | | | | | | ቀልጢፉ ኣብ ሓደ መዓልቲ …………… 1  ቀስ እናበለ ኣብ ኣዋርሕ …………….. 2  ኣይፈልጦን ………………………….. 8 | | | | | | | | |  | | | | | | |  | |
|  | 944 | | | | | | መዋቲ/ት ፍታን እንተይፈተነ/ት ዝወዓለሉ/ትሉ መዓልቲ ወይ ጊዝያት ነይሩ ድዩ? | | | | | | | | እወ …………………………………… 1  ኣይፋሉን ……………………………… 2  ኣይፍለጥን …………………………… 8 | | | | | | | | |  | | | | | | |  | |
|  | 945 | | | | | | መዋቲ/ት ኣብ ከብዱ/ዳ ሕበጥ ነይሩዎ/ዋ ድዩ? | | | | | | | | እወ …………………………………… 1  ኣይፋሉን ……………………………… 2  ኣይፍለጥን …………………………… 8 | | | | | | | | | —**►948**  —**►948** | | | | | | |  | |
|  | 946 | | | | | | እቲ ሕበጥ ንኽንደይ ጊዜ ዝኣክል ኣብ ከብዱ/ዳ ፀኒሑ ነይሩ? | | | | | | | | መዓልቲ ……………………  ወርሒ ………………………..  ኣይፈልጦን ……………................. 98 | | | | | | | | |  | | | | | | |  | |
|  | 947 | | | | | | እቲ ናይ ውሽጢ ከብዲ ሕበጥ ኣበይ ከባቢ እዩ ነይሩ? | | | | | | | | ኣብ የማናይ ጎድኒ ላዕለዋይ ክፍሊ ከብዲ……….1  ኣብ ፀጋማይ ጎድኒ ላዕለዋይ ክፍሊ ከብዲ ……..2  ታሕተዋይ ናይ ከብዲ ክፍሊ …………………..3  ኣብ ኩሉ ናይ ከብዲ ክፍሊ ……………………4  ኣይፈልጦን ……………………………………..8 | | | | | | | | |  | | | | | | |  | |
|  | 948 | | | | | | መዋቲ/ት ደረቕ ምግቢ ክበልዕ/ክትበልዕ እንከሎ/ላ ናይ ምውሓጥ ፀገም ወይ ሕማም ይስመዖ/ዓ'ዶ ነይሩ'ዩ? | | | | | | | | እወ …………………………………… 1  ኣይፋሉን ……………………………… 2  ኣይፍለጥን …………………………… 8 | | | | | | | | | —**►950**  —**►950** | | | | | | |  | |
|  | 949 | | | | | | ደረቕ ምግቢ ክበልዕ/ክትበልዕ እንተሎ/ላ እቲ ናይ ምውሓጥ ፀገም ወይ ሕማም ዝስመዖ/ዓ ዝነበረ ንኽንደይ ጊዜ ፀኒሑ ነይሩ? | | | | | | | | መዓልቲ ……………………….  ወርሒ ………………………..  ኣይፈልጦን …………….................98 | | | | | | | | |  | | | | | | |  | |
|  | 950 | | | | | | መዋቲ/ት ፈሳሲ ክ/ክትውሕጥ እንተሎ/ላ ፀገም ወይ ሕማም ይስመዖ/ዓ'ዶ ነይሩ? | | | | | | | | እወ …………………………………… 1  ኣይፋሉን ……………………………… 2  ኣይፍለጥን …………………………… 8 | | | | | | | | | —**►952**  —**►952** | | | | | | |  | |
|  | 951 | | | | | | ፈሳሲ ክ/ክትውሕጥ እንተሎ/ላ ዝስምዖ/ዓ ዝነበረ ፀገም ወይ ድማ ሕማም ንኽንደይ ጊዜ ፀኒሑ ነይሩ? | | | | | | | | መዓልቲ ……………………….  ወርሒ ………………………..  ኣይፈልጦን …………….................98 | | | | | | | | |  | | | | | | |  | |
|  | 952 | | | | | | መዋቲ/ት ሕማም ርእሲ ነይሩዎ/ዋ'ዶ? | | | | | | | | እወ …………………………………… 1  ኣይፋሉን ……………………………… 2  ኣይፍለጥን …………………………… 8 | | | | | | | | | —**►955**  —**►955** | | | | | | |  | |
|  | 953 | | | | | | እቲ ሕማም ርእሲ ንኽንደይ ጊዜ ፀኒሑ ነይሩ? | | | | | | | | መዓልቲ ……………………….  ወርሒ ………………………..  ኣይፈልጦን …………….................98 | | | | | | | | | | |  | | | | |  | |
|  | 954 | | | | | | እቲ ሕማም ርእሲ ብጣዕሚ ብርቱዕ'ዶ ነይሩ? | | | | | | | | እወ …………………………………… 1  ኣይፋሉን ……………………………… 2  ኣይፍለጥን …………………………… 8 | | | | | | | | | | |  | | | | |  | |
|  | 955 | | | | | | መዋቲ/ት ኣብ ክሳዱ/ዳ ናይ ምንቃፅ ወይ ሕማም ምልክት ነይሩዎ/ዋ ድዩ? | | | | | | | | እወ …………………………………… 1  ኣይፋሉን ……………………………… 2  ኣይፍለጥን …………………………… 8 | | | | | | | | | | | —**►957**  —**►957** | | | | |  | |
|  | 956 | | | | | | እቲ ናይ ክሳድ ምንቃፅ ወይ ምሕማም ንኽንደይ ጊዜ ፀኒሑ ነይሩ? | | | | | | | | መዓልቲ ………………………….  ኣይፍለጥን …………………….......98 | | | | | | | | | | |  | | | | |  | |
|  | 957 | | | | | | መዋቲ/ት ናይ ቀልቢ ምዝባዕ'ዶ ነይሩዎ/ዋ? | | | | | | | | እወ …………………………………… 1  ኣይፋሉን ……………………………… 2  ኣይፍለጥን …………………………… 8 | | | | | | | | | | | —**►960**  —**►960** | | | | |  | |
|  | 958 | | | | | | እቲ ናይ ቀልቢ ምዝባዕ ንኽንደይ ጊዜ ዝኣክል ፀኒሑ ነይሩ? | | | | | | | | መዓልቲ ……………………….  ወርሒ ………………………..  ኣይፈልጦን …………….................98 | | | | | | | | | | |  | | | | |  | |
|  | 959 | | | | | | ኣጀማምራ እቲ ናይ ቀልቢ ምዝባዕ ከመይ ነይሩ? | | | | | | | | ብሃንደበት ………….………………… 1  ቀልጢፉኣብ ሓደ መዓልቲ ………… 2  ቀስ ኢሉ ኣብ ብዙሕ መዓልቲ ……… 3  ኣይፈልጦን ………………………… 8 | | | | | | | | | | |  | | | | |  | |
|  | 960 | | | | | | መዋቲ/ት ዉንኡ/ኣ ስሒቱ/ታ'ዶ ነይሩ/ራ? | | | | | | | | እወ …………………………………… 1  ኣይፋሉን ……………………………… 2  ኣይፍለጥን …………………………… 8 | | | | | | | | | | | —**►963**  —**►963** | | | | |  | |
|  | 961 | | | | | | ንኽንደይ ጊዜ ዝኣክል ዉንኡ/ኣ ስሒቱ/ታ ነይሩ/ራ? | | | | | | | | መዓልቲ ……………………….  ወርሒ ………………………..  ኣይፈልጦን ……………................98 | | | | | | | | | | |  | | | | |  | |
|  | 962 | | | | | | ኣጀማምራ እቲ ዉነ ምስሓት ከመይ ነይሩ? | | | | | | | | ብሃንደበት ………….………………… 1  ቀልጢፉ ኣብ ሓደ መዓልቲ ………… 2  ቀስ ኢሉ ኣብ ብዙሕ መዓልቲ……… 3  ኣይፈልጦን ………………………… 8 | | | | | | | | | | |  | | | | |  | |
|  | 963 | | | | | | መዋቲ/ት ሓያል ምንቅጥቃጥ ኣካል (ዘውድቕ ሕማም ከምዘለዎ/ዋ) ነይርዎ/ዋ ዶ? | | | | | | | | እወ …………………………………… 1  ኣይፋሉን ……………………………… 2  ኣይፍለጥን …………………………… 8 | | | | | | | | | | | —**►965**  —**►965** | | | | |  | |
|  | 964 | | | | | | ንኽንደይ ጊዜ ዝኣክል የንቀጥቅጦ/ጣ ፀኒሑ ነይሩ? | | | | | | | | መዓልቲ ……………………….  ወርሒ ………………………..  ኣይፈልጦን ……………..........…......98 | | | | | | | | | | |  | | | | |  | |
|  | 964.1 | | | | | | ኣብ እዋን ሕማሙ/ማ ንኽንደይ ጊዜ ዝኣክል ኣንቀጥቂጥዎ/ዋ ነይሩ? | | | | | | | | ብቑፅሪ……………………….  ኣይፍለጥን …………………………98 | | | | | | | | |  | | | | |  | | | |
|  | 965 | | | | | | መዋቲ/ት ኣፉ/ፋ ምኽፋት ኣቢዎ/ዋ(ለኺቱ/ታ) 'ዶ ነይሩ? | | | | | | | | እወ …………………………………… 1  ኣይፋሉን ……………………………… 2  ኣይፍለጥን …………………………… 8 | | | | | | | | | —**►967**  —**►967** | | | | |  | | | |
|  | 966 | | | | | | ንኽንደይ እዋን ዝኣክል ኣፉ/ፋ እንተይከፈተ/ት ፀኒሑ/ሓ? | | | | | | | | መዓልቲ ………………………….  ኣይፍለጥን ………………………… 98 | | | | | | | | |  | | | | |  | | | |
|  | 967 | | | | | | መዋቲ/ት ኩሉ ሰውነቱ/ታ ኣንቂፅዎ/ዋ ነይሩ'ዶ? | | | | | | | | እወ …………………………………… 1  ኣይፋሉን ……………………………… 2  ኣይፍለጥን …………………………… 8 | | | | | | | | | —**►969**  —**►969** | | | | |  | | | |
|  | 968 | | | | | | ንኽንደይ እዋን ኩሉ ሰውነቱ/ታ ኣንቂፅዎ/ዋ ፀኒሑ ነይሩ? | | | | | | | | መዓልቲ ………………………….  ኣይፍለጥን ………………………… 98 | | | | | | | | |  | | | | |  | | | |
|  | 969 | | | | | | መዋቲ/ት ሓደ ጎድኑ/ና ለሚሱ'ዶ ነይሩ እዩ? | | | | | | | | እወ …………………………………… 1  ኣይፋሉን ……………………………… 2  ኣይፍለጥን …………………………… 8 | | | | | | | | | —**►972**  —**►972** | | | | |  | | | |
|  | 970 | | | | | | እቲ መልመስቲ ሓደ ጎድኒ ንኽንደይ እዋን ፀኒሑ ነይሩ? | | | | | | | | መዓልቲ ……………………….  ወርሒ ………………………..  ኣይፈልጦን …………….........……...98 | | | | | | | | |  | | | | |  | | | |
|  | 971 | | | | | | ኣጀማምራ እቲመልመስቲ ሓደ ጎድኒ ከመይ ነይሩ? | | | | | | | | ብሃንደበት ………….………………… 1  ቀልጢፉ ኣብ ሓደ መዓልቲ ………… 2  ቀስ እናበለ ኣብ ብዙሕ መዓልቲ ……… 3  ኣይፈልጦን ………………………… 8 | | | | | | | | |  | | | | |  | | | |
|  | 972 | | | | | | መዋቲ/ት ታሕተወት መሓዉሩ/ራ ለሚሶም'ዶ ነይሮም'ዮም? | | | | | | | | እወ …………………………………… 1  ኣይፋሉን ……………………………… 2  ኣይፍለጥን …………………………… 8 | | | | | | | | | —**►975**  —**►975** | | | | |  | | | |
|  | 973 | | | | | | እቲ መልመስቲ ታሕተወት መሓውር ንክንደይ እዋን ፀኒሑ ነይሩ? | | | | | | | | መዓልቲ ……………………….  ወርሒ ………………………..  ኣይፈልጦን ……………....................98 | | | | | | | | |  | | | | |  | | | |
|  | 974 | | | | | | ኣጀማምራ እቲ መልመስቲ ታሕተወት መሓውር ከመይ ነይሩ? | | | | | | | | ብሃንደበት……………. ……………. 1  ቀልጢፉ ኣብ ሓደ መዓልቲ ………… 2  ቀስ ኢሉ/ኣብ ብዙሕ መዓልቲ ……….. 3  ኣይፈልጦን …………………………… 8 | | | | | | | | |  | | | | |  | | | |
|  | 975 | | | | | | መዋቲ/ት ሕብሪ ሽንቱ/ታ ተቐይሩ'ዶ ነይሩ'ዩ? | | | | | | | | እወ …………………………………… 1  ኣይፋሉን ……………………………… 2  ኣይፍለጥን …………………………… 8 | | | | | | | | | —**►977**  —**►977** | | | | |  | | | |
|  | 976 | | | | | | እቲ ለውጢ ሕብሪ ሽንቲ ንኽንደይ እዋን ፀኒሑ ነይሩ? | | | | | | | | መዓልቲ ……………………….  ወርሒ ………………………..  ኣይፈልጦን ……………....................98 | | | | | | | | |  | | | | |  | | | |
|  | 977 | | | | | | ኣፍቲ ናይ መወዳእታ ናይ ሕማሙ/ማ እዋን ሽንቲ መዋቲ/ት ደም ይሓውስ'ዶ ነይሩ እዩ? | | | | | | | | እወ …………………………………… 1  ኣይፋሉን ……………………………… 2  ኣይፍለጥን …………………………… 8 | | | | | | | | | —**►979**  —**►979** | | | | |  | | | |
|  | 978 | | | | | | ንኽንደይ እዋን ዝኣክል እቲ ሽንቲ ደም ይሓውስ ነይሩ/ራ? | | | | | | | | መዓልቲ ……………………….  ወርሒ ………………………..  ኣይፈልጦን ……………..................98 | | | | | | | | |  | | | | |  | | | |
|  | 979 | | | | | | መዋቲ/ት በቢ መዓልቱ ኣብ ዝሸኖ/ትሸኖ ሽንቲ ናይ መጠን ለውጢ ነይሩዎ'ዶ? | | | | | | | | እወ …………………………………… 1  ኣይፋሉን ……………………………… 2  ኣይፍለጥን …………………………… 8 | | | | | | | | | —**►982**  —**►982** | | | | |  | | | |
|  | 980 | | | | | | እቲ ለውጢ መጠን መዓልታዊ ሽንቲ ንኽንደይ እዋን ዝኣክል ፀኒሑ? | | | | | | | | መዓልቲ ……………………….  ወርሒ ………………………..  ኣይፈልጦን ……………..........98 | | | | | | | | |  | | | | |  | | | |
|  | 981 | | | | | | መዋቲ/ት ናይ ሽንቱ/ታ መጠን ብጣዕሚ ብዙሕ ድዩ፣ ብጣዕሚ ንኡሽተይ፣ ወይስ ሽንቲ ኣይሽንን/ኣይትሸንን ነይሩ/ራ? | | | | | | | | ብጣዕሚ ብዙሕ ………………………. 1  ብጣዕሚ ንኡሸተይ …………………… 2  ሽንቲ ኣይሸንን/ኣይትሸንን …………….. 3  ኣይፈልጦን …………………………... 8 | | | | | | | | |  | | | | |  | | | |
|  | 982 | | | | | | መዋቲ/ት ንሞት ዘብቅዖ/ዓ ሕማም ሓሚሙ/ማ እናሃለወ/ት ኣብ ሰውነቱ/ታ ሽፍታ/ዕንፍሩር ነይሩዎ/ዋ'ዶ? | | | | | | | | እወ …………………………………… 1  ኣይፋሉን ……………………………… 2  ኣይፍለጥን …………………………… 8 | | | | | | | | | **►986**  **►986** | | | | |  | | | |
|  | 983 | | | | | | እቲ ሽፍታ/ዕንፍሩር ንኽንደይ እዋን ኣብ ሰውነቱ/ታ ፀኒሑ ነይሩ? | | | | | | | | መዓልቲ……………………….  ኣይፍለጥን ……………………………98 | | | | | | | | |  | | | | |  | | | |
|  | 984 | | | | | | እቲ ሽፍታ/ዕንፍራር ኣበየናይ ክፋል ኣካል ነይሩ   1 ኣብ ገፅ? 2 ኣ ኣፍ ልቢ፣ ከብድን ዝባንን? 3 ኣብ ኢድን እግርን? 4 ኣብ ካልእ ቦታ? | | | | | | | | **እወ ኣይፋል. ኣይፈልጥን**  ገፅ ………………… 1 2 8  ኣፍልቢ፣ ከብዲን ዝባን. 1 2 8  ኢድን እግርን ……. 1 2 8  ካልእ ቦታ …………. 1 2 8  (ይነፀር) _______________ | | | | | | | | |  | | | | |  | | | |
|  | 985 | | | | | | እቲ ሽፍታ/ዕንፍሩር እንታይ ይመስል ነይሩ? | | | | | | | | ናይ ንፍዮ ዕንፍሩር……………………… 1  ፅሩይ ማይ ዝሓዘ ዕንፍሩር……………… 2  መግሊ ዝሓዘ ዕንፍሩር …………………. 3  ኣይፈልጦን …………………………… 8 | | | | | | | | |  | | | | |  | | | |
|  | 986 | | | | | | መዋቲ/ት ዓይኑ/ና ቀይሑ'ዶ ነይሩ? | | | | | | | | እወ …………………………………… 1  ኣይፋሉን ……………………………… 2  ኣይፍለጥን …………………………… 8 | | | | | | | | |  | | | | |  | | | |
|  | 987 | | | | | | መዋቲ/ት መድመይቲ ኣፍንጫ፣ ኣፍ ወይ ከዓ መዓኮር ነይሩዎ/ዋ ድዩ? | | | | | | | | እወ …………………………………… 1  ኣይፋሉን ……………………………… 2  ኣይፍለጥን …………………………… 8 | | | | | | | | |  | | | | |  | | | |
|  | 988 | | | | | | መዋቲ/ት ኣልማዝ ባለጭራ ወፂዎ/ዋ'ዶ ይፈልጥ ነይሩ'ዩ? | | | | | | | | እወ …………………………………… 1  ኣይፋሉን ……………………………… 2  ኣይፍለጥን …………………………… 8 | | | | | | | | |  | | | | |  | | | |
|  | 989 | | | | | | መዋቲት/ት ክብደቱ/ታ ቀኒሱ/ሳ'ዶ ነይሩ/ራ? | | | | | | | | እወ …………………………………… 1  ኣይፋሉን ……………………………… 2  ኣይፍለጥን …………………………… 8 | | | | | | | | | **►990**  **►990** | | | | |  | | | |
|  | 989.1 | | | | | | ንኽንደይ እዋን ዝኣክል ክብደት ቀኒሱ/ሳ ነይሩ/ራ? | | | | | | | | መዓልቲ ……………………….  ወርሒ ………………………..  ኣይፈልጦን ……………..........…....... 98 | | | | | | | | |  | | | | |  | | | |
|  | 989.2 | | | | | | መዋቲ/ት ክረአ/ክትርአ እንተሎ/ላ ቀጢኑ/ና ወይ መንሚኑ/ና'ዶ ነይሩ/ራ? | | | | | | | | እወ …………………………………… 1  ኣይፋሉን ……………………………… 2  ኣይፍለጥን …………………………… 8 | | | | | | | | |  | | | | |  | | | |
|  | 990 | | | | | | መዋቲ/ት ቁስለትኣፍ ፣ ፀዓዱ ነገራት ኣብ ኣፍ ወይ መልሓስ ነይሩዎ/ዋ'ዶ? | | | | | | | | እወ …………………………………… 1  ኣይፋሉን ……………………………… 2  ኣይፍለጥን …………………………… 8 | | | | | | | | | **►991**  **►991** | | | | |  | | | |
|  | 990.1 | | | | | | እቲ ቁስለትኣፍ ፣ ፃዓዱ ነገራት ኣብ ኣፍን መልሓስን ንኽንደይ እዋን ፀኒሑ ነይሩ? | | | | | | | | መዓልቲ……………………….  ኣይፍለጥን …………………………… 98 | | | | | | | | |  | | | | |  | | | |
|  | 991 | | | | | | መዋቲ/ት ዝኾነ ዓይነት ሕበጥ ነይሩዎ/ዋ ድዩ? | | | | | | | | እወ …………………………………… 1  ኣይፋሉን ……………………………… 2  ኣይፍለጥን …………………………… 8 | | | | | | | | | **►992**  **►992** | | | | |  | | | |
|  | 991.1 | | | | | | እቲ ሕበጥ ንኽንደይ እዋን ፀኒሑ ነይሩ? | | | | | | | | መዓልቲ ……………………….  ወርሒ ………………………..  ኣይፈልጦን ……………...................98 | | | | | | | | |  | | | | |  | | | |
|  | 991.2 | | | | | | እቲ ሕበጥ ኣበየናይ ክፋል ኣካል ነይሩ:   1 ገፅ? 2 ኣብ መገጣጠምቲ ኣካላት? 3 ኣብ ዓካሪቶ? 4 ኩሉ ሰውነት? 5 ኣብ ካልእ ቦታ? | | | | | | | | **እወ ኣይፋል. ኣይፈልጥን**  ገፅ ……………… 1 2 8  መገጣጠሚ ……….. 1 2 8  ዓካሪቶ………………….1 2 8  ኩሉ ሰውነት …….... 1 2 8  ካልእ ቦታ …………… 1 2 8  (ይነፀር) _______________ | | | | | | | | |  | | | | |  | | | |
|  | 992 | | | | | | መዋቲ/ት ሕበጥ ፅኪ ነይሩዎ/ዋ? | | | | | | | | እወ …………………………………… 1  ኣይፋሉን ……………………………… 2  ኣይፍለጥን …………………………… 8 | | | | | | | | | **►993**  **►993** | | | | |  | | | |
|  | 992.1 | | | | | | እቲ ሕበጥፅኪ ንኽንደይ እዋን ፀኒሑ ነይሩ? | | | | | | | | መዓልቲ ……………………….  ወርሒ ………………………..  ኣይፈልጦን …………….................. 98 | | | | | | | | |  | | | | |  | | | |
|  | 992.2 | | | | | | እቲ ሕበጥፅኪ ኣበየናይ ክፋል ኣካል ነይሩ?   1 ክሳድ? 2 መንኮራኩዕቲ? 3 ረቃቒቶ? 4 ካልእ ቦታ? | | | | | | | | **እወ ኣይፋል. ኣይፈልጥን**  ክሳድ ……………… 1 2 8  መንኮራኩዕቲ ………… 1 2 8  ረቃቒቶ ………………. 1 2 8  ካልእ ቦታ …………. 1 2 8  (ይገለፅ) _______________ | | | | | | | | |  | | | | |  | | | |
|  | 993 | | | | | | መዋቲ/ት ሕብሪ ዓይኑ/ና ናብ ቢጫ 'ዶ ተቐይሩ ነይሩ'ዩ? | | | | | | | | እወ …………………………………… 1  ኣይፋሉን ……………………………… 2  ኣይፍለጥን …………………………… 8 | | | | | | | | | **►994**  **►994** | | | | |  | | | |
|  | 993.1 | | | | | | መዋቲ/ት ሕብሪ ዓይኑ/ና ቢጫ ዝኾነ ንኽንደይ እዋን እዩ ነይሩ? | | | | | | | | መዓልቲ ……………………….  ወርሒ ………………………..  ኣይፈልጦን …………….................98 | | | | | | | | |  | | | | |  | | | |
|  | 994 | | | | | | መዋቲ/ት መንጪሑ/ሓ (ደም ብምስኣን) ወይ ድማ ከብዲ ኢዱ/ዳ ፣ዓይኑ/ና፣ ፅፍሩን/ራን ፃዕድዩ'ዶ ነይሩ? | | | | | | | | እወ …………………………………… 1  ኣይፋሉን ……………………………… 2  ኣይፍለጥን …………………………… 8 | | | | | | | | | **►995**  **►995** | | | | |  | | | |
|  | 994.1 | | | | | | ንኽንደይ እዋን ዝኣክል መንጪሑ/ሓ ወይ ድማ ከብዲ ኢዱ/ዳ፣ ዓይኑ/ናን ፅፍሩን/ራን ፃዕድዩ ፀኒሑ ነይሩ? | | | | | | | | መዓልቲ ……………………….  ወርሒ ………………………..  ኣይፈልጦን ……………..................98 | | | | | | | | |  | | | | |  | | | |
|  | 995 | | | | | | መዋቲ/ት ኣብ ሰውነቱ/ታ መግሊ ዝሓዘ ሕበጥ ወይ ዝቖሰለ ቦታ ነይሩዎ/ዋ ድዩ? | | | | | | | | እወ …………………………………… 1  ኣይፋሉን ……………………………… 2  ኣይፍለጥን …………………………… 8 | | | | | | | | | **►1001**  **►1001** | | | | |  | | | |
|  | 995.1 | | | | | | እቲ ኣብ ሰውነት ዝነበረ መግሊ ዝሓዘ ሕበጥ ወይ ቑስሊ ንኽንደይ እዋን ፀኒሑ ነይሩ? | | | | | | | | መዓልቲ……………………….  ኣይፍለጥን ……………………….... 98 | | | | | | | | |  | | | | |  | | | |
|  | 995.2 | | | | | | እቲ መግሊ ዝሓዘ ወይ ዝቖሰለ ቦታ ኣበይናይ ክፋል ኣካል እዩ ነይሩ ? | | | | | | | | (ይገለፅ) | | | | | | | | |  | | | | |  | | | |
|  | **ክፍሊ 10 ፡ ነቲ ናይ መወዳእታ ሕማም ዝተገበረ ሕክምናን ኣጠቓቕማ ግልጋሎት ጥዕናን** | | | | | | | | | | | | | | | | | | | | | | | | | | | |  | | | |
|  | 1001 | | | | | | መዋቲ/ት ንሞት ንዘብቅዖ/ዓ ሕማም ተሓኪሙ/ማ'ዶ ነይሩ/ራ? | | | | | | | | | እወ …………………………………… 1  ኣይፋሉን ……………………………… 2  ኣይፍለጥን …………………………… 8 | | | | | | | | | **►1101**  **►1101** | | | |  | | | |
|  | 1002 | | | | | | ንሞት ንዘብቅዖ/ዓ ሕማም ኣበይ ከም ዝተሓከመ/ት ክሕብሩለይ/ራለይ'ዶ?   1. ገዛ? 2. ባህላዊ ሓኪም? 3. መንግስታዊ ጥዕና ኬላ? 4. መንግስታዊ ጥዕና ጣብያ? 5. መንግስታዊ ሆስፒታል? 6. ናይ ውልቀ ክሊኒክ? 7. ናይ ውልቀ ጥዕና ጣብያ? 8. ናይ ውልቀ ሆስፒታል? 9. ፋርማሲ ፣መድሓኒት መሸጢ መደበር? 10. ኣብ ካልእ ቦታ ወይ ትካል? | | | | | | | | | **እወ ኣይፋል. ኣይፈልጥን**  ኣብ ገዛ …………………….1 2 8  ባህላዊ ሕክምና……………..1 2 8  መንግስታዊ ጥዕና ኬላ…......1 2 8  መንግስታዊ ጥዕና ጣብያ…...1 2 8  መንግስታዊ ሆስፒታል……..1 2 8  ውልቀ ክሊኒክ ……………..1 2 8  ውልቀ ጥዕና ጣብያ…………1 2 8  ውልቀ ሆስፒታል…………...1 2 8  ፋርማሲ መድሓኒት መደበር..1 2 8  ካልእ ቦታ _______............1 2 8  (ይገለፅ) | | | | | | | | |  | | | |  | | | |
|  | 1002.1 | | | | | | ናይ ፈለማ ሓገዝ ዝረኸበሉ/ትሉ ኣበይ ነይሩ? | | | | | | | | |  | | | | | | | | |  | | | |  | | | |
|  | 1002.2 | | | | | | ብምቕፃል ንኻልኣይ ጊዜ ሓገዝ ኣበይ ረኺቡ/ባ ነይሩ/ራ? | | | | | | | | |  | | | | | | | | |  | | | |  | | | |
|  | 1002.3 | | | | | | ንሳልሳይ ጊዜ ሓገዝ ኣበይ ረኺቡ/ባ ነይሩ/ራ? | | | | | | | | |  | | | | | | | | |  | | | |  | | | |
|  | 1003 | | | | | | መዋቲ/ቲት ንሞት ዘብቅዖ/ዓ ሕማም ንምፍዋስ ዝተውሃቦ/ባ መድሓኒት ብዝርዝር ዶ ክነግሩኒ/ራኒ?  *(መድሓኒት መአዘዚ ወረቐት/ካብ ጥዕና ትካል ክወፅእ/ክትወፅእ ዝተውሃቦ/ባ መረዳእታ እንተሊዩ ካብኡ ቅዳሕ፡፡)* | | | | | | | | |  | | | | | | | | |  | | | |  | | | |
|  | 1004 | | | | | | እንታይ ዓይነት መድሓኒት ተዋሂብዎ/ዋ:   1 ኦ.ኣር.ኤስ/ወይ ብደም ስር ብመርፍእ ዝውሃብ ፈሳሲ? 2 ደም ምሃብ? 3 በኣፍንጫ ውሽጢ ብትቦ ዝወሃብ ምግቢ ወይ መድሓኒት? 4 ካልእ መድሓኒት? | | | | | | | | | **እወ ኣይፋል. ኣይፈልጥን**  ኦ.ኣር.ኤስ/ብደም ስር ዝወሃብ  መድሓኒት…………………1 2 8  ደም ምሃብ ……………..1 2 8  ብኣፍንጫ ዝውሃብ……….1 2 8  ካልእ መድሓኒት ……….1 2 8  (ይገለፅ)_______________ | | | | | | | | |  | | | |  | | | |
|  | 1005 | | | | | | መዋቲ/ት ቅድሚ ምሟቱ/ታ ኣብ ሓደ ወርሒ ውሽጢ ክንደይ ጊዜ ናብ ጥዕና ትካል ተመላሊሱ/ሳ ነይሩ/ራ? | | | | | | | | | በዝሒ ዝኻደሉ/ትሉ………….  ኣይፈልጦን ……………………… 98 | | | | | | | | |  | | | |  | | | |
|  | 1006 | | | | | | ምኽንያት ሞት እቲ መዋቲ ብበዓል ሞያ ጥዕና ተሓቢሩሎም/ለን'ዶ ነይሩ? | | | | | | | | | እወ …………………………………… 1  ኣይፋሉን ……………………………… 2  ኣይፍለጥን …………………………… 8 | | | | | | | | | **►1008**  **►1008** | | | |  | | | |
|  | 1007 | | | | | | እቲ በዓል ሙያ ጥዕና እንታይ ኢልዎም/ወን ነይሩ? | | | | | | | | |  | | | | | | | | |  | | | |  | | | |
|  | 1008 | | | | | | መዋቲ/ት ንሕማሙ/ማ መጥባሕቲ ተገይርሉ/ሩላ 'ዶ ነይሩ? | | | | | | | | | እወ …………………………………… 1  ኣይፋሉን ……………………………… 2  ኣይፍለጥን …………………………… 8 | | | | | | | | | **►1101**  **►1101** | | | |  | | | |
|  | 1009 | | | | | | እቲ መጥባሕቲ ቅድሚ ምሟቱ/ታ ቅድሚ ክንደይ መዓልቲ እዩ ተገይሩሉ/ላ ነይሩ? | | | | | | | | | መዓልቲ …………………………..  ኣይፈልጦን …………………… 98 | | | | | | | | |  | | | |  | | | |
|  | 1010 | | | | | | እቲ መጥባሕቲ ኣበይናይ ናይ ሰውነቱ/ታ ክፍሊ እዩ ተገይሩ ነይሩ? | | | | | | | | | ከብዲ ……………………………… 1  ደረት ………………………………...... 2  ርእሲ…………………………………… 3  ካልእ _________________ 6  (ይገለፅ)  ኣይፈልጦን ……………………………. 8 | | | | | | | | |  | | | |  | | | |
|  | **ክፍሊ 11 ፡ ንሕማም ዘቃልዑ ተዛመድቲ ነገራት** | | | | | | | | | | | | | | | | | | | | | | | | | | | |  | | | |
|  | 1101 | | | | | | | መዋቲ/ት መስተ ኣልኮል ይሰቲ/ትሰቲ'ዶ ነይሩ/ራ? | | | | | | | እወ …………………………………… 1  ኣይፋሉን ……………………………… 2  ኣይፍለጥን …………………………… 8 | | | | | | | | **►1106**  **►1106** | | | | | |  | | | |
|  | 1102 | | | | | | | ንኽንደይ እዋን ዝኣክል ሰትዩ/ያ?  *(ትሕቲ ሓደ ዓመት እንተኾይኑ 00 መዝግብ/ቢ)* | | | | | | | ዓመት …………………………..  ኣይፈልጦን …………………… 98 | | | | | | | |  | | | | | |  | | | |
|  | 1103 | | | | | | | ክንደይ ዝኣክል ኣዘውቲሮም/ረን ይሰትዩ/ያ ነይሮም/ነይረን? | | | | | | | በቢ መዓልቱ ………………………… 1  ብተደጋጋሚ (ኣብ ሰሙን) ……………… 2  ስሕት ኢሎም/ለን ……………… 3  ኣይፈልጦን ………………………… 8 | | | | | | | |  | | | | | |  | | | |
|  | 1104 | | | | | | | መስተ ምስታይ ኣቋሪፆም/ፀን'ዶ ነይሮም/ረን? | | | | | | | እወ …………………………………… 1  ኣይፋሉን ……………………………… 2  ኣይፍለጥን …………………………… 8 | | | | | | | | **►1106**  **►1106** | | | | | |  | | | |
|  | 1105 | | | | | | | ቅድሚ ምሟቶም/ተን ቅድሚ ክንደይ ጊዜ ኣቀዲሞም/መን መስተ ጠጠው ኣቢሎም/ለን ነይሮም/ረን?  *(ትሕቲ ሓደ ወርሒ እንተኾይኑ 00 መዝግብ/ቢ)* | | | | | | | ኣዋርሕ ………………………..  ኣይፈልጦን …………………… 98 | | | | | | | |  | | | | | |  | | | |
|  | 1106 | | | | | | | መዋቲ/ት ሽጋራ/ትምባኾ ይስሕቡ/ባ'ዶ ነይሮም'ዮም? | | | | | | | እወ …………………………………… 1  ኣይፋሉን ……………………………… 2  ኣይፍለጥን …………………………… 8 | | | | | | | | **►1112**  **►1112** | | | | | |  | | | |
|  | 1107 | | | | | | | ሽጋራ/ትምባኾ ንኽንደይ እዋን ዝኣክል ስሒቦም/በን?  *(ትሕቲ ሓደ ዓመት እንተኾይኑ 00 መዝግብ/ቢ)* | | | | | | | ዓመት ………………………..  ኣይፈልጦን …………………… 98 | | | | | | | |  | | | | | |  | | | |
|  | 1108 | | | | | | | ክንደይ ዝኣክል ኣዘውቲሮም/ረን የትክኹ/ኻ ነይሮም/ረን? | | | | | | | በቢ መዓልቱ ……………………… 1  ብተደጋጋሚ (ኣብ ሰሙን)……………… 2  ስሕት ኢሎም/ለን ……………… 3  ኣይፈልጦን …………………………… 8 | | | | | | | |  | | | | | |  | | | |
|  | 1109 | | | | | | | ኣብ መዓልቲ ክንደይ ሽጋራ ይስሕቡ/ባ ነይሮም/ረን? | | | | | | | ቁፅሪ ሽጋራ……….  ኣይፈልጦን …………………… 98 | | | | | | | |  | | | | | |  | | | |
|  | 1110 | | | | | | | ቅድሚ ምሟቶም/ተን ሽጋራ ምስሓብ ኣቋሪፆም/ፀን'ዶ ነይሮም/ረን? | | | | | | | እወ …………………………………… 1  ኣይፋሉን ……………………………… 2  ኣይፍለጥን …………………………… 8 | | | | | | | | **►1112**  **►1112** | | | | | |  | | | |
|  | 1111 | | | | | | | ቅድሚ ምሟቶም/ተን ቅድሚ ክንደይ እዋን ኣቋሪፆም/ፀን ነይሮም/ረን?  *(ትሕቲ ሓደ ወርሒ እንተኾይኑ 00 መዝግብ/ቢ)* | | | | | | | ኣዋርሕ ………………………..  ኣይፈልጦን …………………… 98 | | | | | | | |  | | | | | |  | | | |
|  | 1112 | | | | | | | መዋቲ/ት ጫት ቁሒሞም/መን ይፈልጡ/ጣ'ዶ? | | | | | | | እወ …………………………………… 1  ኣይፋሉን ……………………………… 2  ኣይፍለጥን …………………………… 8 | | | | | | | | **►1117**  **►1117** | | | | | |  | | | |
|  | 1113 | | | | | | | ንኽንደይ እዋን ዝኣክል ጫት ቁሒሞም/መን ነይሮም/ረን?  *(ትሕቲ ሓደ ዓመት እንተኾይኑ 00 መዝግብ/ቢ)* | | | | | | | ዓመት ………………………..  ኣይፈልጦን …………………… 98 | | | | | | | |  | | | | | |  | | | |
|  | 1114 | | | | | | | ክንደይ ዝኣክል ኣዘውቲሮም/ረን ይቑሕሙ/ማ ነይሮም/ረን? | | | | | | | በቢ መዓልቱ ………………………… 1  ብተደጋጋሚ (ኣብ ሰሙን) ……………… 2  ስሕት ኢሎም/ለን ……………… 3  ኣይፈልጦን …………………………… 8 | | | | | | | |  | | | | | |  | | | |
|  | 1115 | | | | | | | መዋቲ/ት ቅድሚ ምሟቱ/ታ ጫት ምቑሓም ኣቋሪፆም/ፀን'ዶ ነይሮም/ረን? | | | | | | | እወ …………………………………… 1  ኣይፋሉን ……………………………… 2  ኣይፍለጥን …………………………… 8 | | | | | | | | **►1117**  **►1117** | | | | | |  | | | |
|  | 1116 | | | | | | | ቅድሚ ምሟቶም/ተን ቅድሚ ክንደይ እዋን ጫት ምቑሓም ኣቋሪፆም/ፀን ነይሮም/ረን?  ትሕቲ ሓደ ወርሒ እንተኾይኑ 00 መዝግብ/ቢ | | | | | | | ወርሒ ………………………..  ኣይፈልጦን …………………… 98 | | | | | | | | |  | | | | | |  | | |
|  | 1117 | | | | | | | ቃለ መሕትት ዝተወደአሉ ሰዓት መዝግብ/ቢ | | | | | | | ሰዓት  ደቒቓ | | | | | | | | |  | | | | | |  | | |

**ትዕዝብቲ መረዳእታ ኣካባይ**

**ቓለ መሕትት ምስተወደአ ዝምላእ ቕጥዒ**

**______________________________________________________________________________________**______________________________________________________________________________________________________________________________________________________________________________________________________________________________________________________________________________________________________________________________________________________________________________________________________________________________________________

ሪኢቶ ኣብ ሕቶታት

**______________________________________________________________________________________**________________________________________________________________________________________________________________________________________________________________________________________________________________________________________________________________________________________________________________________________________________________

ካልእ ተወሳኺ ሪኢቶ

______________________________________________________________________________________________________________________________________________________________________________________________________________________________________________________________________________________________________________________________________________________________________________________________________________________________________________

ሪኢቶ ተቖፃፃሪ/ት

______________________________________________________________________________________________________________________________________________________________________________________________________________________________________________________________________________________________________________________________________________________________________________________________________________________________________________

__________________________________________________________________________________________________________________________________________________________________________________________________________________________________________________________________

ሽም ተቖፃፃሪ/ት: ___________________________	ዕለት ______________________
